# Tandem DNA repeats contain *cis*‐regulatory sequences that activate biotrophy‐specific expression of *Magnaporthe* effector gene *PWL2*

**DOI:** 10.1111/mpp.13038

**Published:** 2021-03-10

**Authors:** Jie Zhu, Jun Seop Jeong, Chang Hyun Khang

**Affiliations:** ^1^ Department of Plant Biology University of Georgia Athens Georgia USA; ^2^ Department of Biology North Carolina A&T State University Greensboro North Carolina USA; ^3^ Present address: Department of Plant Pathology University of California Davis California USA

**Keywords:** *cis*‐element, gene regulation, *Magnaporthe oryzae*, promoter, rice blast

## Abstract

During plant infection, fungi secrete effector proteins in coordination with distinct infection stages. Thus, the success of plant infection is determined by precise control of effector gene expression. We analysed the *PWL2* effector gene of the rice blast fungus *Magnaporthe oryzae* to understand how effector genes are activated specifically during the early biotrophic stages of rice infection. Here, we used confocal live‐cell imaging of *M. oryzae* transformants with various *PWL2* promoter fragments fused to sensitive green fluorescent protein reporter genes to determine the expression patterns of *PWL2* at the cellular level, together with quantitative reverse transcription PCR analyses at the tissue level. We found *PWL2* expression was coupled with sequential biotrophic invasion of rice cells. *PWL2* expression was induced in the appressorium upon penetration into a living rice cell but greatly declined in the highly branched hyphae when the first‐invaded rice cell was dead. *PWL2* expression then increased again as the hyphae penetrate into living adjacent cells. The expression of *PWL2* required fungal penetration into living plant cells of either host rice or nonhost onion. Deletion and mutagenesis experiments further revealed that the tandem repeats in the *PWL2* promoter contain 12‐base pair sequences required for expression. We conclude that *PWL2* expression is (a) activated by an unknown signal commonly present in living plant cells, (b) specific to biotrophic stages of fungal infection, and (c) requires 12‐base pair *cis*‐regulatory sequences in the promoter.

## Graphical abstract

Expression of the effector gene *PWL2* of the rice blast fungus is controlled by tandem repeat *cis*‐regulatory sequences in response to unknown signals from living cells of host (rice) or non‐host (onion).

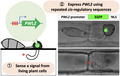

## 1 INTRODUCTION

Fungal infections on susceptible plants are facilitated by hundreds of secreted proteins, collectively known as effectors, that modulate host cell structure, metabolism, and function (Giraldo & Valent, 2013; Sanchez‐Vallet et al., 2018). The success and degree of infection is influenced by the precise control of effector gene expression. There is growing evidence that distinct sets of effector genes are expressed in a coordinated manner in successive waves during the course of infection while these genes are transcriptionally repressed during vegetative growth (Dong et al., 2015; Gervais et al., 2017; Hacquard et al., 2013; Kleemann et al., 2012; Lanver et al., 2018; O’Connell et al., 2012; Tan & Oliver, 2017). In hemibiotrophic pathogens, including the rice blast fungus *Magnaporthe oryzae*, some effector genes, called biotrophy‐specific effector genes, are expressed during a biotrophic stage of infection, and other effector genes are expressed during a later necrotrophic stage of infection (Mosquera et al., 2009; O’Connell et al., 2012). What specific conditions trigger effector gene expression and how the expression is transcriptionally regulated in an infection stage‐specific manner remain largely unknown. Answering these questions has the potential to reveal processes unique to fungal pathogens that can be exploited as novel targets for disease control.

**FIGURE 1:**
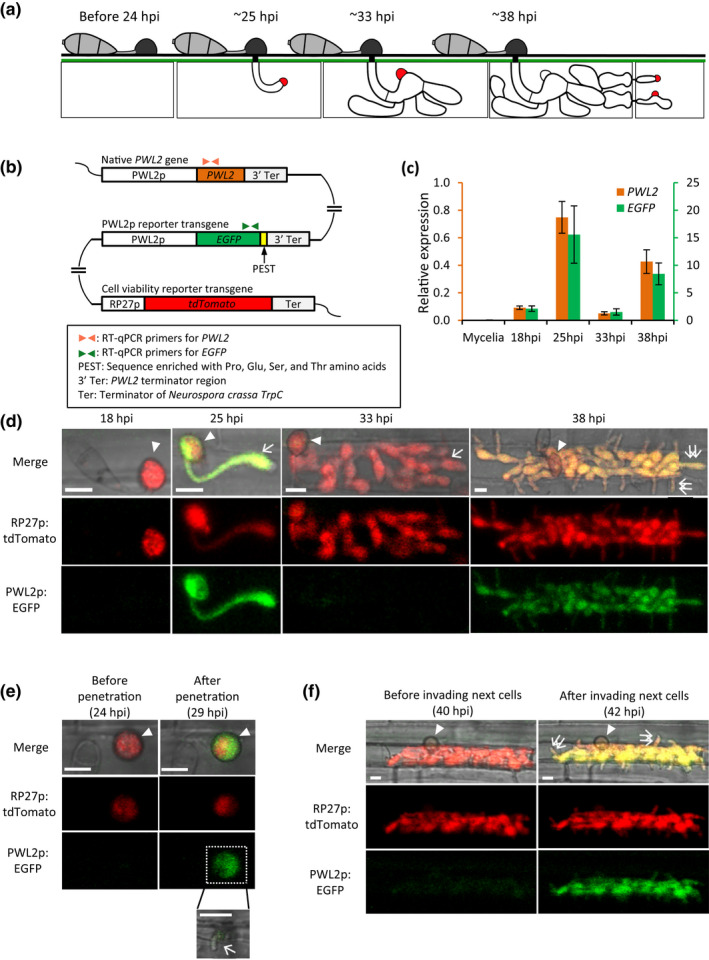
Induced *PWL2* expression occurs during appressorium‐mediated penetration and cell‐to‐cell movement of invasive hyphae (IH). (a) Schematic diagram of *Magnaporthe oryzae* invasion in rice cells. An appressorium on the rice cell surface produces a filamentous hypha that grows inside the living rice cell (c.25 hr postinoculation [hpi]). The hyphal tip is associated with a biotrophic interfacial complex (BIC or tip BIC; indicated in red). The hypha subsequently differentiates into branched bulbous IH, and the tip BIC becomes a side BIC positioned on the side of the first bulbous cell (c.33 hpi). After colonizing the first‐invaded rice cell, IH invade adjacent living cells (c.38 hpi). (b) Schematic diagram of the native *PWL2* gene and two reporter transgenes, enhanced green fluorescent protein (EGFP) under control of the *PWL2* promoter (PWL2p:EGFP) and tdTomato under control of the constitutively active *RP27* promoter (RP27p:tdTomato), inserted ectopically in *M. oryzae* transformant CKF3538. (c) Quantitative reverse transcription PCR expression patterns of native *PWL2* and transgene *EGFP* in CKF3538 with three biological replications. (d) Confocal images of CKF3538 invading rice cells at different stages of infection. (e, f) Time‐lapse confocal images showing the activation of the *PWL2* promoter immediately after appressorium‐mediated penetration (e) and IH cell‐to‐cell movement (f). The inset in (e) shows a short filamentous hypha that grew inside the rice cell. Arrowheads indicate appressoria. Arrows indicate some IH in the first invaded cells, and double arrows indicate some IH that have moved to adjacent cells. Bars, 10 μm

*M. oryzae* causes devastating blast disease in rice and other economically important crops. Recent live‐cell imaging studies, making use of a rice sheath infection assay and various fluorescent reporters, revealed the cellular dynamics associated with the early biotrophic stage of rice infection (Giraldo & Valent, 2013; Jones et al., 2017; Kankanala et al., 2007; Khang et al., 2010; Pfeifer & Khang, 2018; Sakulkoo et al., 2018; Shipman et al., 2017) (Figure 1a). *M. oryzae* biotrophic invasion begins when a single‐celled appressorium produces a penetration peg that breaches the rice cell wall, allowing the fungus to enter a living rice cell. Once inside the first‐invaded rice cell, the penetration peg expands to form a filamentous primary hypha. As the primary hypha switches from filamentous to depolarized growth, the nucleus in the appressorium begins mitosis, and one nucleus undergoes a long‐distance migration to the swollen tip of the primary hypha, followed by a septation, to produce the first bulbous invasive hyphal cell (Shipman et al., 2017). The bulbous invasive hyphae (IH) continue colonizing the first‐invaded host cell for 8–12 hr before moving into adjacent cells using IH pegs that co‐opt plasmodesmata (Kankanala et al., 2007; Sakulkoo et al., 2018). The first‐invaded rice cell is alive but dies when the fungus penetrates adjacent living cells, establishing a pattern of successive biotrophic invasion (Jones et al., 2017; Kankanala et al., 2007). *M. oryzae* secretes biotrophy‐specific effector proteins, including PWL2, into the biotrophic interfacial complex (BIC) presumed to mediate effector translocation into host cells (Khang et al., 2010). The BIC first appears at the tip of the primary hypha (tip‐BIC) but then is repositioned to the side of the first bulbous cell (side‐BIC) in the first‐ and subsequently invaded cells (Khang et al., 2010; Shipman et al., 2017) (Figure 1a). *PWL2* is one of the well‐characterized blast effector genes. *PWL2* was initially cloned as an avirulence gene, which prevented the fungus from infecting weeping lovegrass, and it belongs to a multigene family present in various host‐adapted isolates of *M. oryzae* (Kang et al., 1995; Sweigard et al., 1995). Previous studies have demonstrated that *PWL2* is highly expressed during infection, while its expression is rarely detectable in axenically grown cultures (Mosquera et al., 2009; Nishimura et al., 2016; Sweigard et al., 1995).

In this study, we analysed the *PWL2* gene to understand how this and other effector genes are activated specifically during the early biotrophic invasion of rice cells. The *PWL2* expression pattern and promoter activity were determined using confocal live‐cell imaging of *M. oryzae* transformants with various *PWL2* promoter fragments fused to sensitive green fluorescent protein (GFP) reporter genes (destabilized or nuclear‐targeted), together with time‐course quantitative reverse transcription PCR (RT‐qPCR) analyses. We found that *PWL2* expression was coupled with sequential biotrophic invasion and that the expression required fungal penetration into living plant cells of either host rice or nonhost onion. Deletion and mutagenesis experiments further revealed that the tandem repeats in the *PWL2* promoter contain 12‐base pair (bp) sequences required for expression. Taken together, these results show that biotrophy‐specific *PWL2* expression is activated by an unknown signal commonly present in living plant cells and requires 12‐bp *cis*‐regulatory sequences in the promoter.

## 2 RESULTS

### 2.1 Development of *PWL2* promoter reporter strains of *M. oryzae*

To determine the *PWL2* expression pattern, we generated a transcriptional reporter construct by fusing the *PWL2* promoter (PWL2p, 872 bp) and 3′ terminator region (3' ter, 500 bp), respectively, at the 5′ and 3′ ends of a reporter gene that encodes the destabilized version of enhanced green fluorescent protein (EGFP). The rapid turnover of destabilized EGFP allows tracking of the transient increase and decrease of gene expression in living cells (Li et al., 1998). The resulting construct (PWL2p:EGFP) was introduced into an *M. oryzae* strain, constitutively expressing tdTomato under control of the *M. oryzae* ribosomal protein *RP27* promoter (RP27p:tdTomato) (Figure 1b). Our initial confocal imaging of 10 randomly selected transformants consistently showed bright EGFP fluorescence mainly in IH growing inside rice cells, compared to tdTomato fluorescence in all developmental stages of *M. oryzae* (Figure S1). We identified one transformant (*M. oryzae* CKF3538) that showed brighter fluorescence than others. We used RT‐qPCR to determine the expression patterns of the native *PWL2* gene and the ectopically inserted *EGFP* under control of the *PWL2* promoter in this strain (Figure 1b). We found that both the native *PWL2* gene and the *EGFP* transgene (PWL2p:EGFP) displayed a similar pattern of expression. In mycelia from axenic culture, expression of both the native *PWL2* gene and the *EGFP* transgene was not detectable (Figure 1c). During rice infection, expression of both genes peaked at 25 and 38 hours postinoculation (hpi) with lower, or basal, expression detected at 18 and 33 hpi (Figure 1c). We also noticed that the overall transcription level was higher for the *EGFP* transgene than the native *PWL2* gene, probably due to a position effect of the transgene integration into the genome. The consistent expression pattern of the native *PWL2* gene and the *EGFP* transgene confirmed that the *PWL2* promoter reporter strain CKF3538 (PWL2p:EGFP and RP27p:tdTomato) can be used to monitor the transient transcriptional induction of *PWL2* in real time during infection.

### 2.2 Induced expression of *PWL2* during appressorium‐mediated penetration and hyphal cell‐to‐cell movement

To determine the expression pattern of *PWL2* at the cellular level, we used confocal microscopy of *M. oryzae* transformant CKF3538 (PWL2p:EGFP and RP27p:tdTomato) invading living rice cells. EGFP fluorescence (PWL2p:EGFP) was barely detectable in mature appressoria that had not yet penetrated rice cells (18 hpi, *n* = 66). However, EGFP fluorescence was strongly detected in appressoria that had penetrated rice cells and in subsequently growing young IH (25 hpi, *n* = 178). As the fungus continued to grow in the first‐invaded rice cell, EGFP fluorescence declined to a barely detectable level (33 hpi, *n* = 46) but strongly increased again in IH that had spread into adjacent cells (38 hpi, *n* = 36). We confirmed that lack of strong EGFP fluorescence at 18 and 33 hpi was not due to artifacts related to fungal cell death because there was consistent tdTomato fluorescence (RP27p:tdTomato) in all fungal cells (Figures 1d and S1). The expression pattern of EGFP fluorescence (PWL2p:EGFP) was consistent with the RT‐qPCR results (Figure 1c). These results suggest that activation of the *PWL2* promoter coincides with the timing of fungal penetrations into the first rice cells and adjacent rice cells. Consistent with this finding, we observed EGFP fluorescence in the appressorium that produced very short filamentous primary IH (c.11 μm) but not in the proximally located appressorium that had not yet penetrated the first rice cell (Figure S2). Using time‐lapse confocal imaging, we further demonstrated the transition from absence of EGFP fluorescence to strong EGFP fluorescence after the fungus penetrated rice cells and produced even c.10 μm of IH in the first invaded cell (Figure 1e) or 4–8 μm of IH in adjacent rice cells (Figure 1f). Taken together, our results revealed that induction of *PWL2* expression repeatedly occurs immediately after appressorium‐mediated penetration and hyphal cell‐to‐cell movement.

### 2.3 Induction of *PWL2* expression requires penetration into living plant cells

**FIGURE 2:**
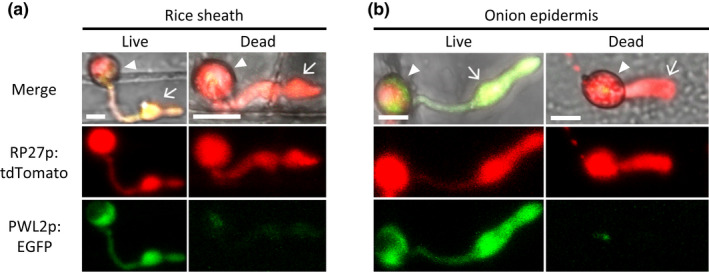
Confocal images of *Magnaporthe oryzae* transformant CKF3538 invading rice cells (a) and nonhost onion cells (b). Note the strong expression of *PWL2* promoter‐driven enhanced green fluorescent protein (EGFP) in living cells but not in dead cells of both rice and onion. The EGFP expression in dead cells was barely detectable even with highly sensitive confocal imaging settings, as shown in Figure S3. Arrowheads indicate appressoria. Arrows indicate invasive hyphae. Bars, 10 μm

Rice cells that are initially invaded by young biotrophic hyphae are viable, but the invaded rice cells subsequently lose viability when the hyphae are fully expanded (Jones et al., 2016, 2017). Given that *PWL2* was expressed as the fungus penetrated into presumed living rice cells (25 and 38 hpi), but the expression was greatly reduced in the fully expanded hyphae in presumed dead rice cells (33 hpi) (Figure 1d), we hypothesized that living rice cells are required for *PWL2* expression. To test this hypothesis, we examined EGFP expression using confocal microscopy of CKF3538 (PWL2p:EGFP and RP27p:tdTomato) inoculated on a heat‐killed rice leaf sheath. After the fungus penetrated heat‐killed rice cells, EGFP fluorescence was barely detectable, which was in stark contrast to strong EGFP fluorescence when the fungus penetrated living rice cells, while tdTomato fluorescence was comparable in both conditions (*n* = 42; Figures 2a and S3a).

To determine whether the induced *PWL2* expression was specific to the host rice plant, we tested *PWL2* expression using an onion peel penetration assay (Xu et al., 1997). Onion is not a natural host of *M. oryzae*, but the fungus penetrates onion epidermal cells using the appressorium. We observed similar results to those in heat‐killed rice cells, that is, EGFP fluorescence (PWL2p:EGFP) was strongly detected when the fungus penetrated living onion cells, whereas the fluorescence was barely detectable when it penetrated heat‐killed onion cells even observed at saturated fluorescence levels (Figures 2b and S3b). We concluded that highly induced *PWL2* expression requires penetration into living plant cells, and the induction is not host specific.

### 2.4 Tandem repeats are required for *PWL2* expression

**FIGURE 3:**
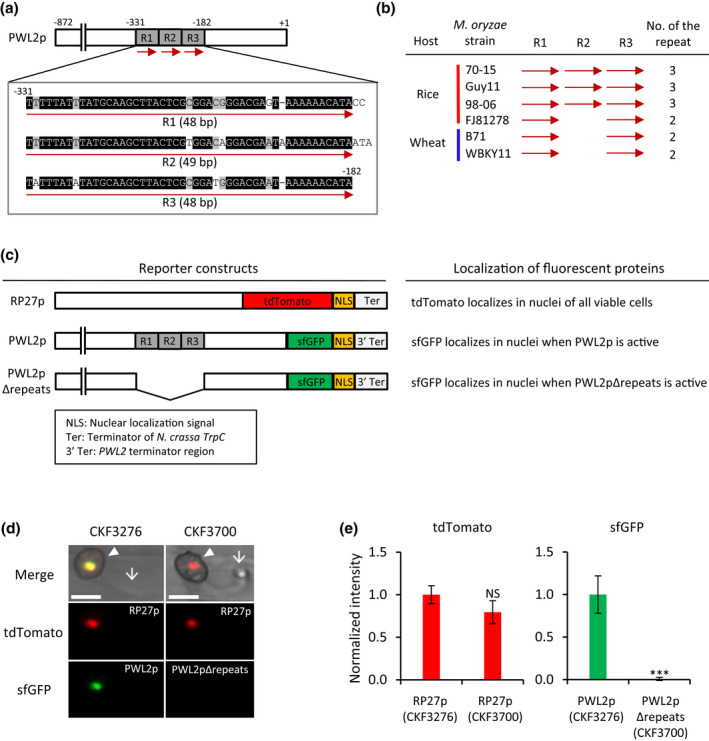
The tandem repeat sequences are required for *PWL2* induction during plant infection. (a) The organization and DNA sequences of the three tandem repeats in the *PWL2* promoter (PWL2p). The repeats are denoted as R1, R2, and R3 with red arrows. The numbers indicate the nucleotide positions relative to the translation start site. Black shading indicates sequences conserved in all three repeats, and grey shading indicates sequences conserved in two repeats. (b) Comparison of the repeat copy number in the *PWL2* promoters of six different *Magnaporthe oryzae* strains, including four rice‐pathogenic strains (red line) and two wheat‐pathogenic strains (blue line). Red arrows indicate the repeats. DNA sequence alignment of the repeats are shown in Figure S4. (c) A schematic representation of the reporter constructs and expected localization of the florescent proteins (red tdTomato and green sfGFP). RP27p is a constitutive promoter. PWL2p is the *PWL2* promoter, and PWL2pΔrepeats is the *PWL2* promoter with deletion of all three tandem repeats. (d) Confocal images of *M. oryzae* transformants expressing sfGFP:NLS (green) under control of PWL2p (*M. oryzae* CKF3276) or PWL2pΔrepeats (*M. oryzae* CKF3700) at 25 hr postinoculation (hpi). Both transformants constitutively express tdTomato:NLS (red) as a control of visualizing nuclei of viable fungal cells. Arrowheads and arrows indicate, respectively, appressoria and filamentous invasive hyphae (IH) that just penetrated rice cells. Bars, 10 μm. (e) Comparison of normalized fluorescence intensities of tdTomato or sfGFP quantified from nuclei of CKF3276 and CKF3700 at 25 hpi. Data are presented as mean ± *SD* of more than 10 infection sites for each strain. Two‐tailed Student's *t* test was performed to determine statistical difference. ***p < .001, NS, no significant difference

The *PWL2* promoter contains three imperfect repeat sequences whose role in transcriptional regulation was not yet studied (Sweigard et al., 1995). These repeats are located between −331 and −182 nucleotides relative to the translation start site, and occur three times in tandem, and thus are named R1 (48 bp), R2 (49 bp), and R3 (48 bp) in this study (Figure 3a). R1 shares 92% sequence identity with R2 or R3, and R2 shares 88% identity with R3 (Figure 3a). From the NCBI database, we identified DNA sequences of the *PWL2* locus in the genome sequences of *M. oryzae* strains adapted to rice or wheat. Sequence comparison revealed that the promoter regions are highly conserved across the strains, but the copy number of the repeats varied, ranging from two to three copies (Figures 3b and S4).

To determine the role of the tandem repeats in *PWL2* expression, we compared the transcriptional activity of the *PWL2* promoter (PWL2p) and the *PWL2* promoter with deletion of all three tandem repeats (PWL2pΔrepeats). Each promoter was fused to a fast‐folding superfolder GFP with a nuclear localization signal (sfGFP:NLS reporter). This construct permitted precise detection and quantification of the intensity of sfGFP fluorescence localized in nuclei when the *PWL2* promoter was activated. Each reporter construct (PWL2p:sfGFP:NLS or PWL2pΔrepeats:sfGFP:NLS) was introduced into the *M. oryzae* strain constitutively expressing tdTomato fused to NLS as a control for visualizing nuclei of viable fungal cells (RP27p:tdTomato:NLS) (Figures 3c and S5). Using confocal microscopy, we first demonstrated that sfGFP fluorescence of the PWL2p:sfGFP:NLS reporter strain (CKF3276) strongly accumulated in the nucleus of the appressorium upon penetration (Figures 3d and S5). We also observed that the fluorescence intensity of this construct decreased in multibranched IH within first‐invaded cells but then increased again when the hyphae moved into adjacent cells (Figure S5). This pattern of increase, decrease, and subsequent increase of *PWL2* promoter activity was consistent with the data generated by RT‐qPCR and another promoter reporter construct (PWL2p:EGFP) (Figure 1c,d,e,f). Given that *PWL2* promoter activity changed during the course of infection, we compared fluorescence intensities of sfGFP driven by PWL2p or PWL2pΔrepeats at two comparable infection stages, specifically focusing on the appressorium that produced young IH (less than two hyphal cells) within the first‐infected rice cell and in IH that spread into adjacent cells. Our confocal imaging clearly showed that there was no detectable sfGFP fluorescence in the PWL2pΔrepeats:sfGFP:NLS strain (CKF3700) at both infection stages while tdTomato fluorescence driven by RP27p in this strain was as strong as that in the PWL2p:sfGFP:NLS strain (CKF3276) (Figures 3d,e and S6). These results suggest that the tandem repeats contain a positive regulatory element required for activation of *PWL2* expression during penetration into living rice cells.

### 2.5 The tandem repeats in the *PWL2* promoter contain *cis*‐regulatory sequences

**FIGURE 4:**
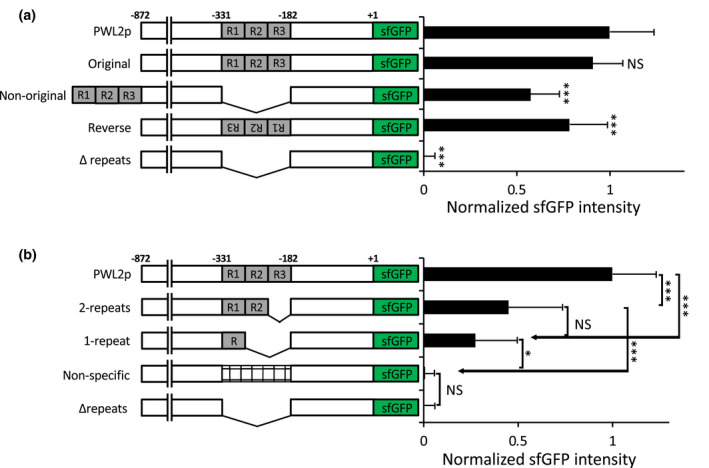
Comparison of promoter activity of a series of *PWL2* promoter constructs fused to the sfGFP:NLS reporter. The three tandem repeats in the *PWL2* promoter are denoted as R1, R2, and R3. The numbers indicate the nucleotide positions relative to the translation start site. The promoter activity was measured by quantifying sfGFP fluorescence in nuclei of *Magnaporthe oryzae* transformants expressing each construct as described for Figure 3c–e. More than two fungal transformants were randomly chosen for each construct, and at least 10 independent infection sites of each transformant were analysed. The native *PWL2* promoter is indicated as PWL2p, and the promoter with all three repeats deleted as Δrepeats. (a) The repeats were inserted back into the original location (Original) or at 500 bp upstream from the original location (Non‐original) or in the reversed orientation at the original location (Reverse) in the repeat‐deleted promoter. Data are presented as mean ± *SD*. Statistically significant differences were determined using Dunnett's test with PWL2p as the control. ***p < .001, NS, no significant difference. (b) Two copies of the repeats were inserted at the original location (2‐repeats) in the repeat‐deleted promoter. One copy of the repeat (1‐repeat), of which DNA sequence is shown in Figure 5a, or a random DNA sequence (Non‐specific) was inserted at the original location in the repeat‐deleted promoter. Data are presented as mean ± *SD*. Statistically significant differences were determined using Tukey–Kramer HSD test: ***p < .001, NS, no significant difference

**FIGURE 5:**
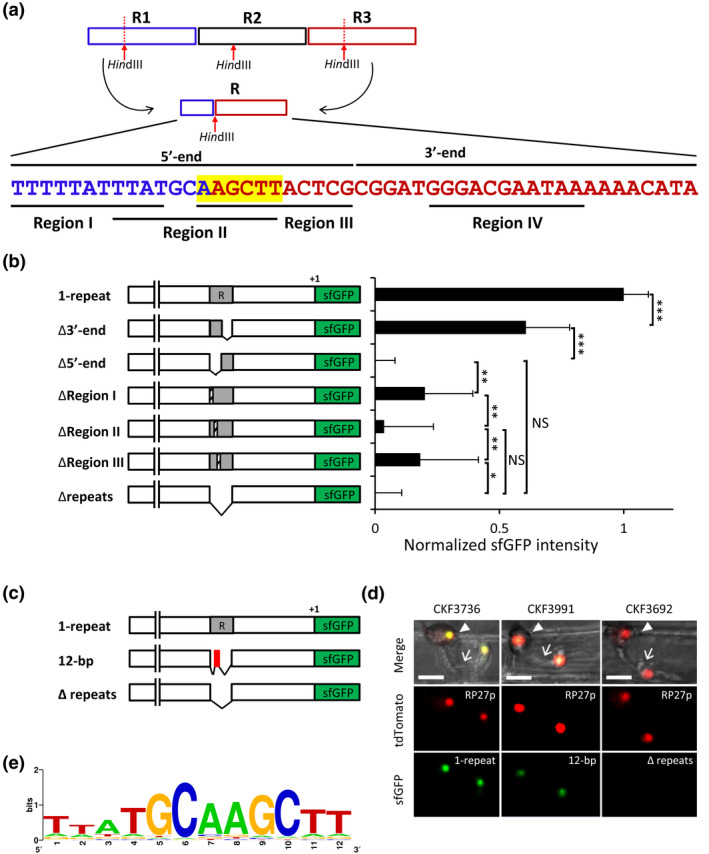
The 12‐bp motif in the tandem repeat sequence is essential for *PWL2* promoter activity. (a) Schematic diagram of generating the *PWL2* promoter with one repeat (R) by *Hin*dIII (AAGCTT) digestion and subsequent ligation to join the 5′ end region of the R1 (14 bp) and the 3′ end of the R3 (34 bp). The first 24 bp of the R is defined as the 5′‐end, and the other 24 bp as the 3′‐end. The 5′‐end was further defined as Region I (11 bp), Region II (12 bp), and Region III (11 bp). (b) The *PWL2* promoter activity with each of the regions defined in (a) being deleted was determined, along with the promoter with one repeat (1‐repeat R generated from Figure 5a and the promoter with no repeat [Δrepeats], as described for Figure 3c–e). More than two fungal transformants were randomly chosen for each construct, and at least 10 independent infection sites of each transformant were analysed. Data are presented as mean ± *SD*. Statistically significant differences were determined by the Tukey–Kramer HSD test. *p < .05, **p < .01, ***p < .001; NS, no significant difference at *p* < .05. (c) Schematic diagram of the *PWL2* promoter with one repeat (1‐repeat) or the 12‐bp motif in place of the repeat (12‐bp; red box corresponding to Region II in Figure 5a) or no repeat (Δrepeats) fused to the sfGFP:NLS reporter. (d) Confocal images of *Magnaporthe oryzae* transformants expressing sfGFP:NLS (green) under control of 1‐repeat (*M. oryzae* CKF3736) or 12‐bp (*M. oryzae* CKF3991) or Δrepeats (*M. oryzae* CKF3692) at 25 hr postinoculation. All transformants constitutively express tdTomato:NLS (red) as a control of visualizing nuclei of viable fungal cells. Arrowheads and arrows indicate, respectively, appressoria and filamentous invasive hyphae (IH) that just penetrated rice cells. Note that there are two nuclei (one in the appressorium and another one in the IH cell). More than five independent infection sites were observed for each of 11 random transformants for the 12‐bp construct, and all showed the consistent result. Bars, 10 μm. (e) Consensus 12‐bp motif sequence generated from 126 sequences similar to the 12‐bp motif shown in Figure 5a using WebLogo (Crooks et al., 2004)

To determine if the *PWL2* promoter activity is affected by a change of location or orientation of the tandem repeats in relation to the translation start site, we made a series of promoter constructs by inserting the repeat sequences back into the repeat‐deleted promoter (Figure 4). These constructs were individually linked to the sfGFP:NLS reporter, and the promoter activity was measured by quantifying sfGFP fluorescence in the resulting transgenic *M. oryzae* strains during appressorium‐mediated penetration, as described above for Figure 3c–e. We first confirmed that when the three copies of the repeats were inserted back into the original location, the promoter activity was fully restored (Original in Figure 4a). Next, we found that insertion of the repeats at a location 500 bp upstream from the original location (Non‐original in Figure 4a) or in the reversed orientation at the original location (Reverse in Figure 4a) resulted in restoration of the promoter activity, although the expression was reduced when compared to the wild‐type *PWL2* promoter (reduction of c.50% or c.25%, respectively; Figure 4a). We further showed that the restoration of the promoter activity was specific to the repeats because when a random DNA of the same length as the repeats was inserted into the repeat‐deleted promoter, there was no sfGFP fluorescence (Non‐specific in Figure 4b). These results suggested that the repeats contain a *cis*‐regulatory element controlling inducibility of the *PWL2* promoter and also that the regulatory activity is not strictly dependent on the location or orientation of the repeats relative to the translation start site. Furthermore, we found that constructs with three repeats showed higher inducibility of the promoter compared to one or two repeats, suggesting that increasing the number of repeats increases promoter strength (Figure 4b). It is important to note that one repeat, designated as R, was generated by taking advantage of three *Hin*dIII (AAGCTT) sites, present in all three repeats at conserved locations. All three *Hin*dIII sites were digested and subsequently ligated to join the 5′ end region of the R1 repeat (14 bp) and the 3′ end of the R3 repeat (34 bp) (Figure 5a). The resulting single‐copy repeat was sufficient for transcription, indicating the presence of a *cis*‐regulatory element within each repeat.

### 2.6 Identification of *cis*‐regulatory sequences in the tandem repeat of the *PWL2* promoter

Our initial BLAST search revealed that the repeat sequences in the *PWL2* promoter shared some similarity with those in the upstream regions of other *M. oryzae* effector and candidate effector genes (Table S1). These shared sequences were short, ranging from 10 to 25 bp long, which we mapped on the single copy of the repeat and defined as Regions I to IV (Figures 5a and S8a). Regions I, II, and III are located within the first 24 bp (5′‐end, Figure 5a), and Region IV is located at the 3′ end of the repeat (3′‐end, Figure 5a). We used a sfGFP:NLS reporter to quantify promoter activity for a series of deletion or substitution mutations in the repeat. We first determined that deletion of the 5′ end (positions 1–24), but not the 3′ end (positions 25–48), completely abolished promoter activity (Figure 5a,b). This suggests the presence of a *cis*‐regulatory element at the 5′ end. To further define the *cis*‐regulatory element, we focused our fine‐scale deletion and mutation analyses on Regions I, II, and III located at the 5′ end. We found that transversion substitution mutations in Regions I (11 bp), II (12 bp), or III (11 bp) reduced promoter activity, and particularly mutations in Region II resulted in the greatest reduction in promoter activity (Figures 5b and S8a). These results suggest that the 12‐bp motif in Region II (5′‐TTATGCAAGCTT‐3′) is a *cis*‐regulatory sequence. This finding was further supported because inserting the 12‐bp motif back into the *PWL2* promoter with the deletion of all three tandem repeats (PWL2pΔrepeats) restored promoter activity (Figure 5c,d).

### 2.7 The 12‐bp‐like motif is present in the upstream region of *M. oryzae* effector genes

We sought to determine if the 12‐bp motif we found in Region II was present in the upstream regions of other *M. oryzae* effector genes. We first identified 540 predicted effector genes in the *M. oryzae* genome using EffectorP v. 1.0 (Sperschneider et al., 2016) and subsequently conducted motif scanning for the original 12‐bp motif and for similar 12‐bp motifs, that is, “12‐bp‐like motifs” within the 1‐kb upstream regions of these genes using MEME suite (Grant et al., 2011). We found a total of 126 occurrences of the 12‐bp or 12‐bp‐like motif (*p* < .0001) in the upstream sequences of 106 genes (19.6% of a total of 540 genes), and in some of these regions the motif occurs more than once (Table S2). The motif‐containing genes include some known effector genes, such as *AVR‐Pik* (MGG_15972), *BAS4* (MGG_10914), *MAX* (MGG_08414), *MAX* (MGG_09425), *MOCDIP3* (MGG_07986), *MoHEG9* (MGG_00043), and *SPD10* (MGG_11991) (Chen et al., 2013; de Guillen et al., 2015; Mogga et al., 2016; Mosquera et al., 2009; Sharpee et al., 2017; Yoshida et al., 2009) (Table S2). A comparison of all 126 motif sequences suggests that the core sequence of the motif is 5′‐TGCAAGCTT‐3′ (Figure 5e).

**FIGURE 6:**
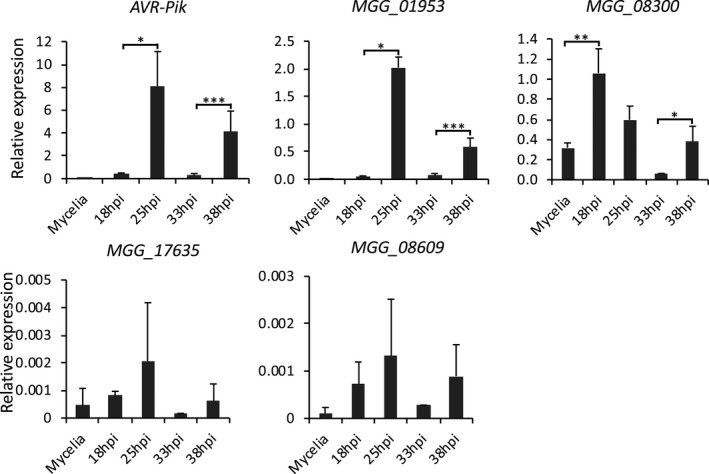
Quantitative reverse transcription PCR analyses of one known effector gene (*AVR‐Pik*) and four candidate effector genes, which all contain the 12‐bp‐like motif in their promoters, show that the expression patterns of these genes are similar to that of *PWL2* (Figure 1c) during axenic culture (mycelia) and infection; hpi, hours postinoculation. Data are presented as mean ± *SD*. Two‐tailed Student's *t* test was performed to determine statistical difference. *p < .05, **p < .01, ***p < .001

To determine if the motif‐containing effector genes are coexpressed with *PWL2*, we used a time‐course RT‐qPCR analysis for 10 selected genes that contain the 12‐bp‐like motif in the same orientation and at a similar location (−200 to −350 bp relative to the translation start site) as in the *PWL2* promoter. We found that five genes showed similar expression patterns to *PWL2* (Figures 1c and 6). In particular, the expression patterns of *AVR‐Pik* and two predicted effector genes (MGG_01953 and MGG_08300) were strikingly similar to that of *PWL2*, exhibiting initial induction at 25 hpi during appressorium‐mediated penetration into living cells and repression at 33 hpi when the fungus colonizes first‐invaded dead cells, followed by subsequent reinduction at 38 hpi when the fungus penetrates into living adjacent cells during cell‐to‐cell movement (Figures 1c and 6). These results indicate that the 12‐bp‐like motif plays a role in regulating the biotrophy‐specific expression of *M. oryzae* effector genes.

## 3 DISCUSSION

### 3.1 *PWL2* expression is coupled with sequential biotrophic invasion

In this study, we provide evidence that transcriptional regulation of *PWL2* is coupled to the biotrophic phase of *M. oryzae* during colonization of the first two rice cells. In *M. oryzae*, biotrophy is characterized as sequential invasion into living rice cells, which begins with the appressorium initially penetrating a living rice cell (c.25 hpi), and then when highly branched IH penetrate adjacent living cells after colonizing the first‐invaded cell until cell death (c.38 hpi) (Figure 1a) (Jones et al., 2017; Kankanala et al., 2007). Our time‐course RT‐qPCR analysis at the tissue level and EGFP reporter‐based live‐cell imaging at single‐cell resolution consistently showed that *PWL2* expression is induced immediately upon penetrating living rice cells from the appressorium or from IH moving to adjacent living rice cells (Figure 1c,d). This strong expression was in stark contrast to the barely detectable expression in the appressorium on the rice cell surface (prior to penetration into the first cell), and highly branched IH in the first‐invaded dead rice cell (prior to penetration into the second cell). This two‐peaked expression pattern of *PWL2* coincides with localization of the PWL2 protein in BICs, which form when the fungus penetrates new living rice cells (Khang et al., 2010; Shipman et al., 2017). These results suggest that *PWL2* transcription is tightly regulated in coordination with BIC development during biotrophic invasion. Shipman et al. (2017) made an intriguing observation that cytoplasmic effector proteins, including PWL2, are secreted into the tip BIC and the early side BIC, which can be located more than 32 μm away from the nearest nucleus in the appressorium where this study shows *PWL2* promoter is activated. It remains to be determined how effector trafficking is regulated from the appressorium through the primary hypha to the distantly located BIC.

### 3.2 *PWL2* expression is activated by an unknown signal in living plant cells

Fungal genes that are induced during infection are transcriptionally regulated presumably in response to a combination of nutrition conditions, plant‐derived inducing compounds, or infection‐related fungal development (Basse et al., 2000; Meyer et al., 2017; Van den Ackerveken et al., 1994; van der Does et al., 2008). Earlier studies by Sweigard et al. (1995) showed that *PWL2* transcripts were not detectable when *M. oryzae* was grown in complete, minimal, or nitrogen‐depleted medium, thus excluding these nutrition conditions as a cue to induce *PWL2* expression. Our finding that *PWL2* expression was induced when the fungus penetrated living cells, but not dead cells, of both rice (host) and onion (nonhost) suggests that the presumed inducer is commonly present in living plant cells (Figure 2). Similar observations have been reported for biotrophy‐specific effector genes in other fungi. For instance, expression of the effector gene *Six1* of *Fusarium oxysporum* is induced upon penetration of the root cortex of living tomato (host) and also in response to cell cultures of tomato and tobacco (nonhost) (van der Does et al., 2008). The plant signals that induce *Six1* expression remain unknown. The effector gene *MiSSP7* of *Laccaria bicolor* is expressed during interactions with poplar (host) and *Arabidopsis thaliana* (nonhost) roots, and the inducer has been identified as rutin and quercetin, commonly found flavonoids in the exudates of plant roots (Plett et al., 2011). Future studies will be needed to identify inducers and regulatory components for *PWL2*, which can be facilitated by using strategies such as mutagenesis and screening plant compounds using GFP‐based reporters (Basse et al., 2000, 2002).

### 3.3 Tandem DNA repeats within the *PWL2* promoter contain *cis*‐regulatory sequences

Tandem DNA repeat sequences are often associated with gene promoters and function as *cis*‐regulatory sequences. For example, tandem repeats are found in 25% of all promoters in the *Saccharomyces cerevisiae* genome (Vinces et al., 2009). Some tandem repeats directly regulate expression of genes such as the maltose permease gene in *S. cerevisiae* (Bell et al., 1997) and the anthocyanin‐regulating transcription factor *MYB10* in apple (Espley et al., 2009). Approximately 52% of the *M. oryzae* genome consists of various repetitive sequences, and a genome‐wide analysis is needed to determine how many *M. oryzae* gene promoters, particularly effector gene promoters, are associated with tandem repeats (Raffaele & Kamoun, 2012). The *PWL2* promoter contains three 48‐bp imperfect tandem repeats (Figure 3a) (Sweigard et al., 1995). Using dot plot analyses, we found that promoters of eight additional effector genes beside *PWL2* contain tandem repeats in various numbers and lengths in their promoters (Figure S9). There is growing evidence that tandem repeat‐containing promoters show higher transcriptional divergence (Vinces et al., 2009). In agreement with this, we found that the inducibility of the *PWL2* promoter varies depending on the copy number of the repeats within the promoter (Figure 4b). Intriguingly, this appears to be further implicated with the role *PWL2* plays during plant infection. *PWL2* confers avirulence against weeping lovegrass, containing a yet‐to‐be discovered resistance gene. *PWL2* is also presumed to have a virulence role in other plants, lacking the resistance gene, based on the prevalent occurrence of *PWL2* in diverse *M. oryzae* populations (Kang et al., 1995; Sweigard et al., 1995). While characterizing DNA sequences required for *PWL2* avirulence activity, Sweigard et al. (1995) showed that avirulence was partially lost when the promoter was deleted to contain one and a half copies of the repeat or completely lost when deleted further to contain the half copy. In contrast, avirulence was fully retained with more than two and a half copies of the repeat. This impaired avirulence is probably due to the reduced transcription of *PWL2* that we observed with the reduced copy number of the repeats (Figure 4b). It is possible that a certain level of *PWL2* transcription, correlated with protein production, is required for the PWL2 protein being recognized as an avirulence factor. We suggest that fine‐tuned expression of *PWL2* through variations in the repeat copies could be a potential mechanism by which *PWL2* avoids host recognition (losing avirulence activity) while retaining a presumed virulence function, thereby facilitating *M. oryzae* host adaptation. Consistent with this notion, we found that different host‐adapted *M. oryzae* strains carry a varying copy number of the repeat in the *PWL2* promoter (Figure 3b). It will be exciting to investigate how repeat copy number variations correlate with *PWL2* expression in these strains and how these variations in copy number contribute to *M. oryzae* adaptibility on different plant species.

We provide evidence that the tandem DNA repeats contain *cis*‐regulatory sequences required for biotrophy‐specific expression of *PWL2*. Deletion of these repeats resulted in a complete loss of *PWL2* expression, which could be complemented when the repeats were inserted at the original location (Figure 4a). The complementation was specific to the sequences of the repeats because an unrelated DNA sequence in the same length as the repeats failed to complement (Figure 4a). We also determined that the single copy of the repeat contained all sequences sufficient for *PWL2* expression (Figure 4b). These data suggest that each repeat contains *cis*‐regulatory sequences, directly regulating gene expression. These *cis*‐regulatory sequences are presumed to function as a transcription factor (TF) binding site rather than as a structural component of the promoter. The location and orientation of TF binding sites can have effect on promoter activity (Lis & Walther, 2016; Sharon et al., 2012). Consistent with these findings, we observed some level of promoter activity even when these repeats were inserted at a distal location or in reverse orientation (Figure 4a). Further deletion and mutagenesis studies identified a 12‐bp motif that is present within each repeat and is sufficient for *PWL2* expression (Figure 5). Addition of the 12‐bp motif to PWL2pΔrepeats restored the GFP reporter expression upon appressorium‐mediated penetration into living rice cells (Figure 5c,d). The 12‐bp‐like motif appears to be involved in regulating expression of other effector genes in *M. oryzae*. We found that at least 106 effector or effector candidate genes contain the 12‐bp or 12‐bp‐like motif in their promoter regions, and five of these genes indeed showed similar expression patterns to *PWL2* (Figure 6). These results suggest that they are transcriptionally co‐regulated by common transcription factors (Lanver et al., 2018). Whether the motif directly regulates expression of these and other genes containing the motif remains to be determined.

Evidence is accumulating that distinct sets of effector genes are coordinately expressed in successive waves during the course of host infection, reflecting the complexity of effector gene regulation and the diversity of TFs and *cis*‐regulatory sequences (Dong et al., 2015; Farfsing et al., 2005; Gervais et al., 2017; Hacquard et al., 2013; Kleemann et al., 2012; Lanver et al., 2018; O’Connell et al., 2012; Soyer et al., 2014; Wang et al., 2011). Fungal genomes collectively contain at least 36 different families of TFs, and 13 TFs from four families are known for their roles in effector regulation (Lin et al., 2018; Tan & Oliver, 2017). *M. oryzae* is predicted to contain a total of 495 TFs (4.5% of the 11,054 proteins in *M. oryzae*) (Park et al., 2013), and thus far MoGti1 is the only TF known to regulate the expression of *M. oryzae* effector genes, including *PWL2* (Li et al., 2016). The precise mechanism of how MoGti1 controls transcription of these effector genes is not known. Our hypothesis is that MoGti1 has an indirect role in activating *PWL2* transcription based on the fact that the 12‐bp motif or the rest of the *PWL2* promoter lacks the core binding motif (5′‐TTAAAGTTT‐3′), recognized by an MoGti1 ortholog, Wor1, in *Candida albicans* (Lohse et al., 2010). Our discovery of the 12‐bp motif provides exciting opportunities for testing this hypothesis, and also predicting new effector candidates based on the presence of the motif in their promoters.

## 4 EXPERIMENTAL PROCEDURES

### 4.1 Strains, fungal transformation, and plasmid construction

*M. oryzae* wild‐type strain O‐137 was used as a recipient strain to generate fungal transformants using *Agrobacterium tumefaciens*‐mediated transformation (Khang et al., 2005). *M. oryzae* strains were cultured on oatmeal agar plates at 24 °C under continuous light and stored frozen at −20 °C to maintain full pathogenicity (Valent et al., 1991). See Tables S3 and S4 for the list of *M. oryzae* strains and plasmids used in this study. To monitor *PWL2* expression at single‐cell resolution, *M. oryzae* transformant CKF3538 was made by sequential transformation of pCK1292 and pCK1714 into wild‐type strain O‐137. pCK1292 was generated by cloning a 0.5‐kb *Eco*RI‐*Bam*HI fragment of pBV167 (*RP27* promoter) (Shipman et al., 2017) and 1.7‐kb *Bam*HI‐*Hin*dIII fragment of pAN582 (pBV359, tdTomato:Nos terminator) (Nelson et al., 2007) in *Eco*RI‐*Hin*dIII sites of pBV141 (pBGt) (Kim et al., 2011). pCK1714 was generated by cloning a 1.7‐kb *Eco*RI*‐Bsr*GI fragment of pCK1298 (PWL2p:EGFP), a 0.12‐kb *Bsr*GI‐*Not*I fragment of pBV118 (pd2EGFP‐1) (Li et al., 1998), and a 0.5‐kb *Not*I‐*Xho*I fragment of pBV1102 (*PWL2* 3′ terminator region) in *Eco*RI‐*Sal*I sites of pBV1 (pBHt2) (Mullins et al., 2001).

To identify the *cis*‐element in the *PWL2* promoter, fungal transformants with fluorescently labelled nuclei were generated. In particular, *M. oryzae* transformant CKF3276 was generated by sequential transformation of pCK1528 and pCK1586 into wild‐type strain O‐137. pCK1528 was generated by cloning a 1.0‐kb *Eco*RI*‐Bam*HI fragment of pBV126 (*RP27* promoter) (Khang et al., 2010), a 1.4‐kb *Bam*HI*‐Bsr*GI fragment of pAN582 (tdTomato:NLS), and a 0.4‐kb *Bsr*GI‐*Hin*dIII fragment of pBV578 (Nos terminator) in *Eco*RI*‐Hin*dIII sites of pBV141. pCK1586 was generated by cloning a 872‐bp *Eco*RI*‐Bam*HI fragment of pCK1574 (*PWL2* promoter) and a 1.3‐kb *Bam*HI‐*Xho*I fragment of pCK1576 (sfGFP without ATG:NLS:*PWL2* 3′ terminator region) in *Eco*RI‐*Sal*I sites of pBV1. *sfGFP* was first cloned from sfGFP‐Lifeact‐7 (pCK1349), a gift from Michael Davidson (Addgene plasmid # 54739). A series of constructs with deletions, replacements, and mutations in the *PWL2* promoter was generated by restriction enzyme digestion and ligation, or PCR using the primers in Table S5 (Figures S8 and S9). The intact or manipulated *PWL2* promoter fragments and sfGFP:NLS:PWL2 3′ terminator region were first cloned into pJET1.2 (Thermo Fisher Scientific) to confirm the introduction of the desired alteration by DNA sequencing, and later the fragments were inserted into the binary vector pBV1.

After two rounds of selections on TB3 (0.3% yeast extract, 0.3% casamino acid, 20% sucrose) and V8 (8% V8 vegetable juice [Campbell's], pH 7) media containing 200 µg/ml of hygromycin (Fisher BioReagents) or 800 µg/ml of G418 (Fisher BioReagents), and 200 µM of cefotaxime (Gold Biotechnology), at least 10 independent transformants for each construct were selected and purified by single spore isolation. All positive transformants showed similar fluorescence patterns and behaved indistinguishably when compared to the wild‐type strain under microscope examination. Due to the position effect of transgenic genes (Chen & Zhang, 2016; Soanes et al., 2002), two or three independent transformants for each construct were randomly chosen for further analysis.

### 4.2 Infection assays

Rice sheath inoculations were performed using the susceptible rice YT16 as previously described (Jones & Khang, 2018). Briefly, excised leaf sheaths (5–8 cm long) from 19‐ to 21‐day old plants were inoculated with a spore suspension (10^5^ spores/ml in distilled water). The inoculated sheaths were hand‐trimmed and immediately used for confocal microscopy. Inoculations on onion epidermal peels were performed as previously described (Xu et al., 1997). In heat‐killed inoculations, pretrimmed sheaths or onion epidermal peels were incubated in 70 °C water for 25 min, cooled down to room temperature, and then inoculated with a spore suspension.

### 4.3 RNA isolation and RT‐qPCR

For the RT‐qPCR assay, 15 infected rice sheaths at each time point of 18, 25, 33, and 38 hpi were collected as described (Mosquera et al., 2009), frozen immediately in liquid nitrogen, and stored at −80 °C for RNA extraction. The TRIzol method (Invitrogen) was used to extract total RNA. Genomic DNA was eliminated by Turbo DNase (Ambion) according to the manufacturer's instructions. Complementary DNA (cDNA) was synthesized using the ImProm II Reverse Transcriptase system (Promega) from 500 ng of total RNA extracted from infected tissue or mycelia grown for 5 days in complete medium (CM). RT‐qPCR was performed with the MX3005P (Stratagene) systems using the VeriQuest SYBR Green qPCR Master Mix (2×; Thermo Fisher). Each reaction (final volume 14 µl) contained 7 µl of VeriQuest SYBR Green qPCR Master Mix, 1.5 µl of each the forward and reverse primers (3.3 nM concentrations for each, Table S5), 2 µl of cDNA, and 2 µl of distilled water. Thermocycler conditions were as follows: 2 min at 50 °C, 10 min at 95 °C; followed by 40 cycles of 95 °C for 30 s, 60 °C for 30 s, and 72 °C for 30 s. The specificity of each primer pair was checked by incorporation of a final dissociation cycle. The relative expression level was calculated using the 2^−∆*C*t^ method (Livak & Schmittgen, 2001) with the *M. oryzae actin* gene (MGG_03982) as the reference gene (Che Omar et al., 2016). Briefly, the average threshold cycle (*C*_t_) was normalized to that of the *actin* gene for each sample as 2^‐Δ*C*t^, where Δ*C*_t_ = (*C*_t, target gene_ − C_t, actin_). Two technical replications for each of three biological replications were performed. Mean and standard deviation were calculated from three biological replicates.

### 4.4 Confocal microscopy and quantification of fluorescence intensity

Confocal microscopy was performed on Zeiss LSM 510 Meta and Zeiss LSM 880 confocal microscopes. Excitation/emission wavelengths were 488 nm/496–544 nm for EGFP/sfGFP and 543 nm/565–617 nm for tdTomato. Images were processed using Zen Black software v. 10.0 (Zeiss). To quantify and compare fluorescence intensity for each experiment, pinhole size and detector gain were optimized to maximize detection range to avoid saturated pixels. Identical image acquisition and processing settings were used to analyse all images for each experiment. Quantification of fluorescence intensity was performed using ImageJ (https://imagej.nih.gov/ij/) as previously described (Hartig, 2013; Jensen, 2013). Briefly, an image was first maximum‐intensity projected and split into different channels. The image threshold was adjusted until all fluorescent areas were selected. Intensity measurements were then performed for each nucleus. *PWL2* promoter activity was defined as normalized sfGFP intensity from 0 to 1, with 0 being background intensity as determined in nuclei without fluorescence, and 1 being the highest intensity value in nuclei of the fungal transformant with the 872‐bp *PWL2* promoter as determined by subtraction of background intensity.

### 4.5 Sequence analysis and effector prediction

DNA sequence analyses were performed using Geneious software v. 8.1.2 (https://www.geneious.com/) with sequences obtained from the NCBI database. The 1,000‐bp upstream sequences of *M. oryzae* genes were obtained from the Broad Institute (https://www.broadinstitute.org/). The sequences were then imported into Geneious and used as the database for a BLASTn analysis. The 12‐bp motif was used for a motif occurrence search by FIMO (MEME v. 4.12.0, http://meme‐suite.org/tools/fimo) with default parameters (Grant et al., 2011) against the 1,000‐bp upstream sequences of *M. oryzae* effector genes. The alignment of *PWL2* sequences was conducted using ClustalW (Thompson et al., 2003) implemented in Geneious. The previously generated *M. oryzae* secretome data (Zhang et al., 2018) were used to predict effector genes using EffectorP v. 1.0 (Sperschneider et al., 2016).

## CONFLICT OF INTEREST

The authors declare that they have no conflict of interest.

## Supporting information


**FIGURE S1** *PWL2* expression is induced during fungal invasion inside of rice cells but not in axenically grown cultures

**FIGURE S2** *PWL2* expression is only observed in the appressorium of penetrating epidermal cells of the rice sheath at 25 hr postinoculation

**FIGURE S3** Confocal images of *Magnaporthe oryzae* transformant CKF3538 invading rice cells and nonhost onion cells with a highly sensitive setting

**FIGURE S4** Sequence alignment of the promoter regions of *PWL2* genes from various *Magnaporthe oryzae* isolates

**FIGURE S5** Nucleus‐localized fluorescent reporter with the *PWL2* promoter shows a consistent *PWL2* expression pattern

**FIGURE S6** The tandem repeats are required for *PWL2* induction during cell‐to‐cell movement of invasive hyphae

**FIGURE S7** Graphic presentation of the *PWL2* promoter regions and modified tandem repeats, and the sfGFP fusion constructs used in this study

**FIGURE S8** Graphic presentation of the deletions and substitutions of one repeat, and the sfGFP fusion constructs used in this study

**FIGURE S9** The promoter regions of multiple effector genes in *Magnaporthe oryzae* have repeat sequences

**TABLE S1** Effector and effector candidate genes identified by BLAST search of the tandem repeat R1 in promoters of *Magnaporthe oryzae* genes

**TABLE S2** Predicted effector genes with a 12‐bp‐like motif in promoters

**TABLE S3** *Magnaporthe oryzae* strains used in this study

**TABLE S4** Key plasmids used in this study

**TABLE S5** PCR primers used in this study

## Data Availability

The data that support the findings of this study are available from the corresponding author upon reasonable request.
