# Supplementary material for: Tandem DNA repeats contain cis‐regulatory sequences that activate biotrophy‐specific expression of Magnaporthe effector gene PWL2
Source: Mol Plant Pathol. 2021 Mar 10;22(5):508–21. doi: 10.1111/mpp.13038 (PMC8035637; doi:10.1111/mpp.13038)
Supplement: Supplementary file 1 — FIGURE S1 PWL2 expression is induced during fungal invasion inside of rice cells but not in axenically grown cultures [file MPP-22-508-s011.pptx]

## Slide 1
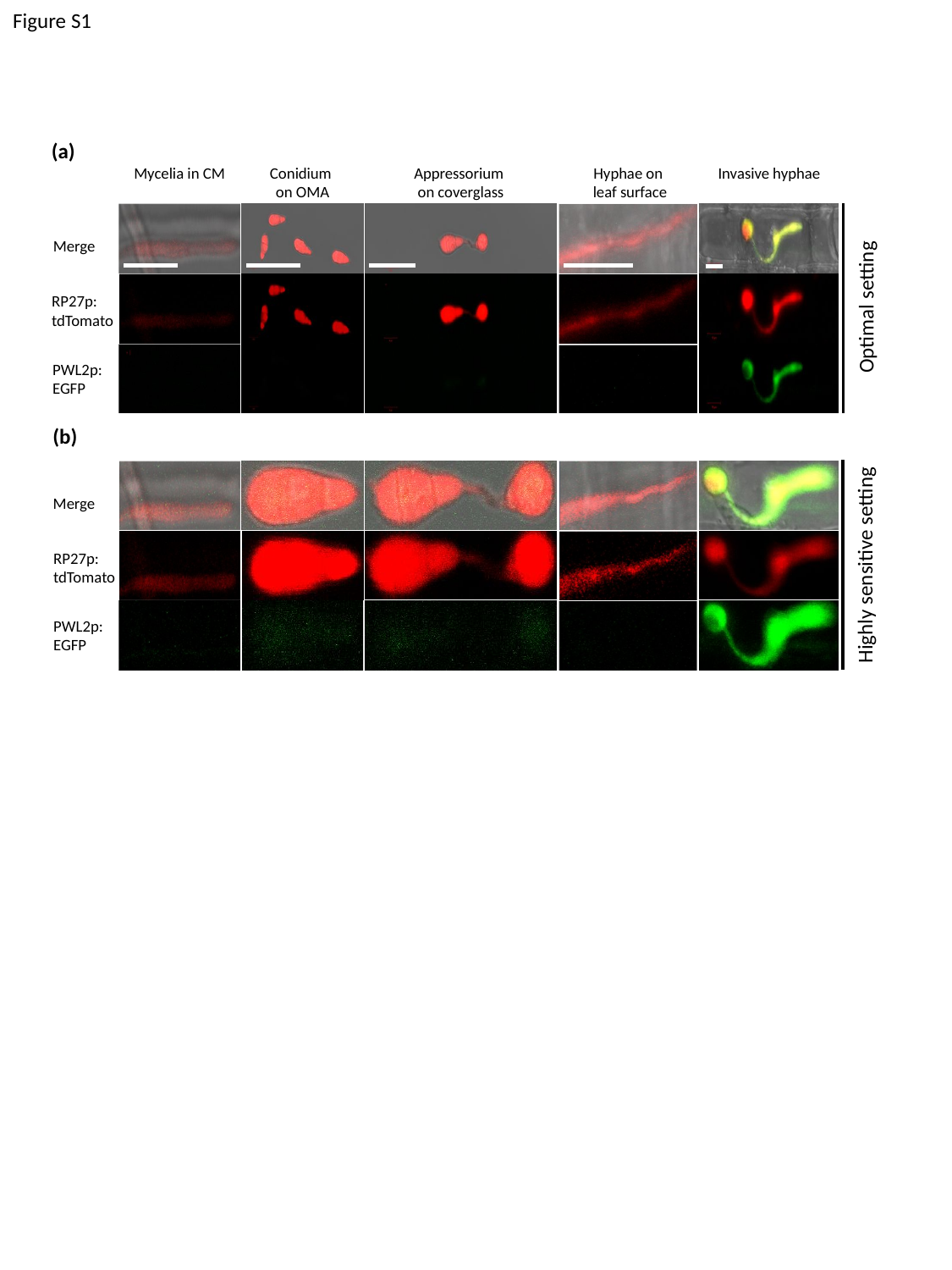

Figure S1
(a)
Hyphae on
 leaf surface
Appressorium
on coverglass
Conidium
on OMA
Mycelia in CM
Invasive hyphae
Merge
RP27p:
tdTomato
Optimal setting
PWL2p:
EGFP
(b)
Merge
RP27p:
tdTomato
Highly sensitive setting
PWL2p:
EGFP
