# Supplementary figures and images for: Tandem DNA repeats contain cis‐regulatory sequences that activate biotrophy‐specific expression of Magnaporthe effector gene PWL2
Source: Mol Plant Pathol. 2021 Mar 10;22(5):508–21. doi: 10.1111/mpp.13038 (PMC8035637; doi:10.1111/mpp.13038)

## Slide 1
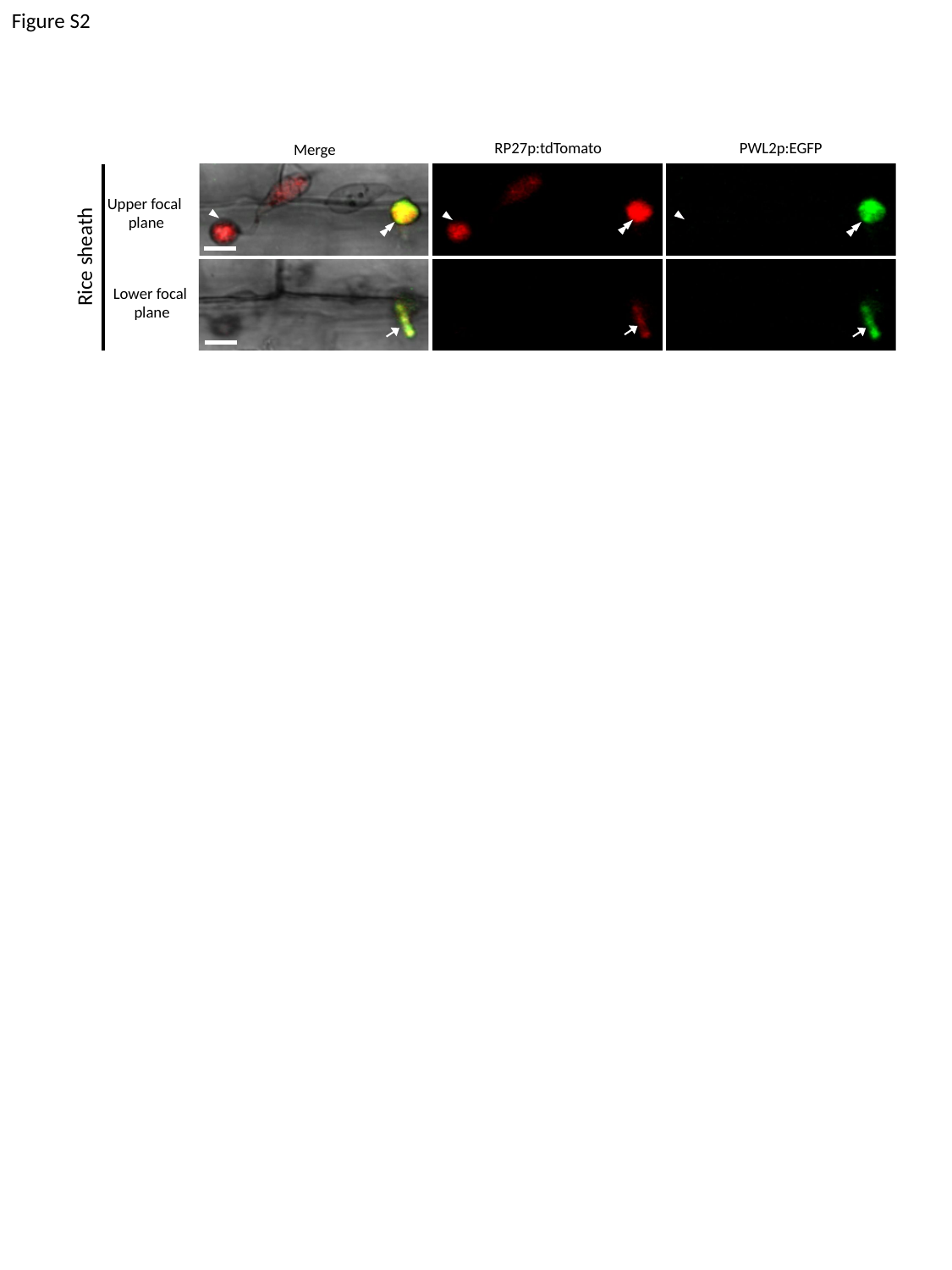

Figure S2
RP27p:tdTomato
PWL2p:EGFP
Merge
Upper focal
plane
Rice sheath
Lower focal
plane

Supplement: Supplementary file 2 — FIGURE S2 PWL2 expression is only observed in the appressorium of penetrating epidermal cells of the rice sheath at 25 hr postinoculation [file MPP-22-508-s008.pptx]

## Slide 1
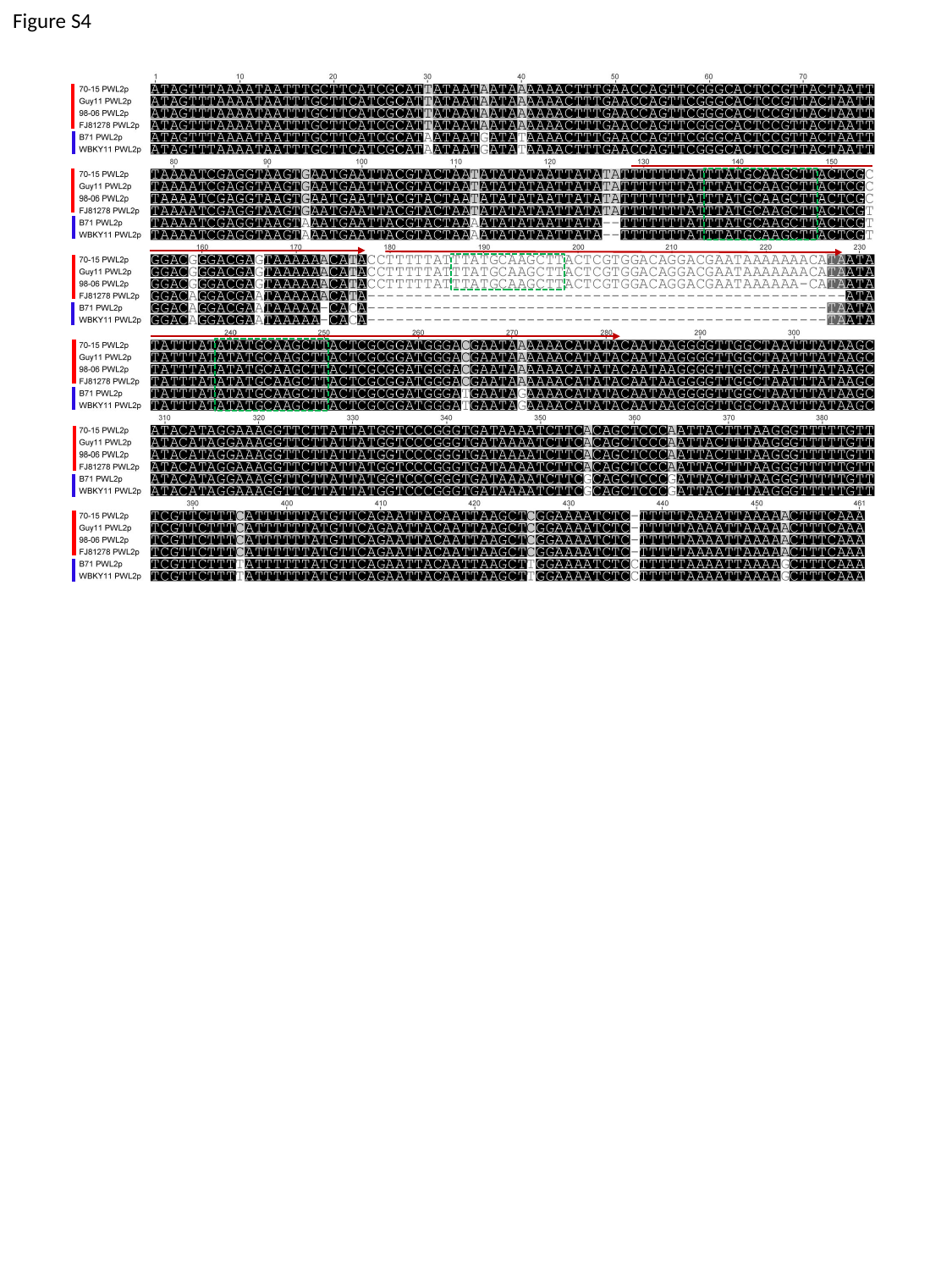

Figure S4

Supplement: Supplementary file 4 — FIGURE S4 Sequence alignment of the promoter regions of PWL2 genes from various Magnaporthe oryzae isolates [file MPP-22-508-s012.pptx]

## Slide 1
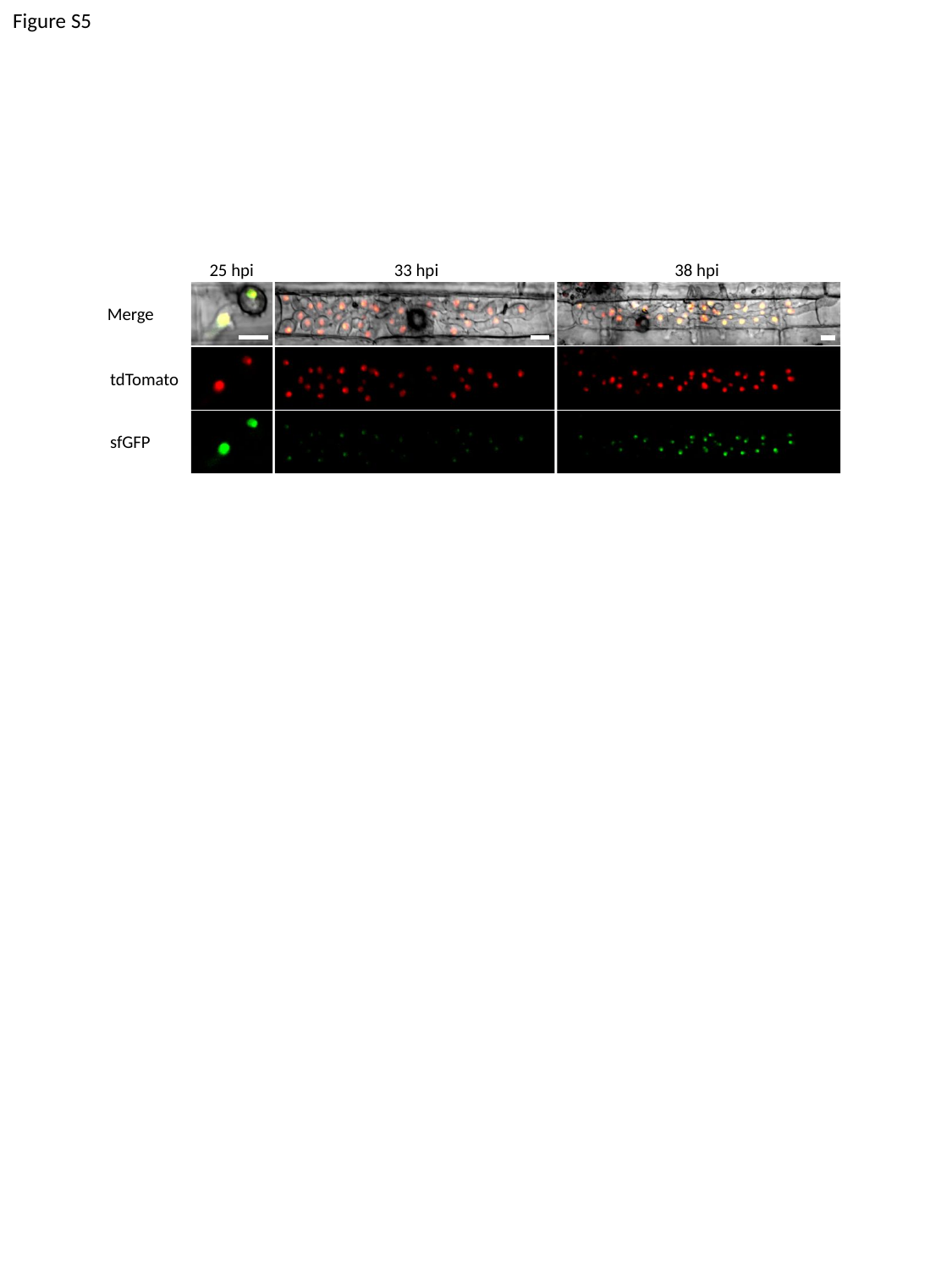

Figure S5
33 hpi
38 hpi
25 hpi
Merge
tdTomato
sfGFP

Supplement: Supplementary file 5 — FIGURE S5 Nucleus‐localized fluorescent reporter with the PWL2 promoter shows a consistent PWL2 expression pattern [file MPP-22-508-s007.pptx]

## Slide 1
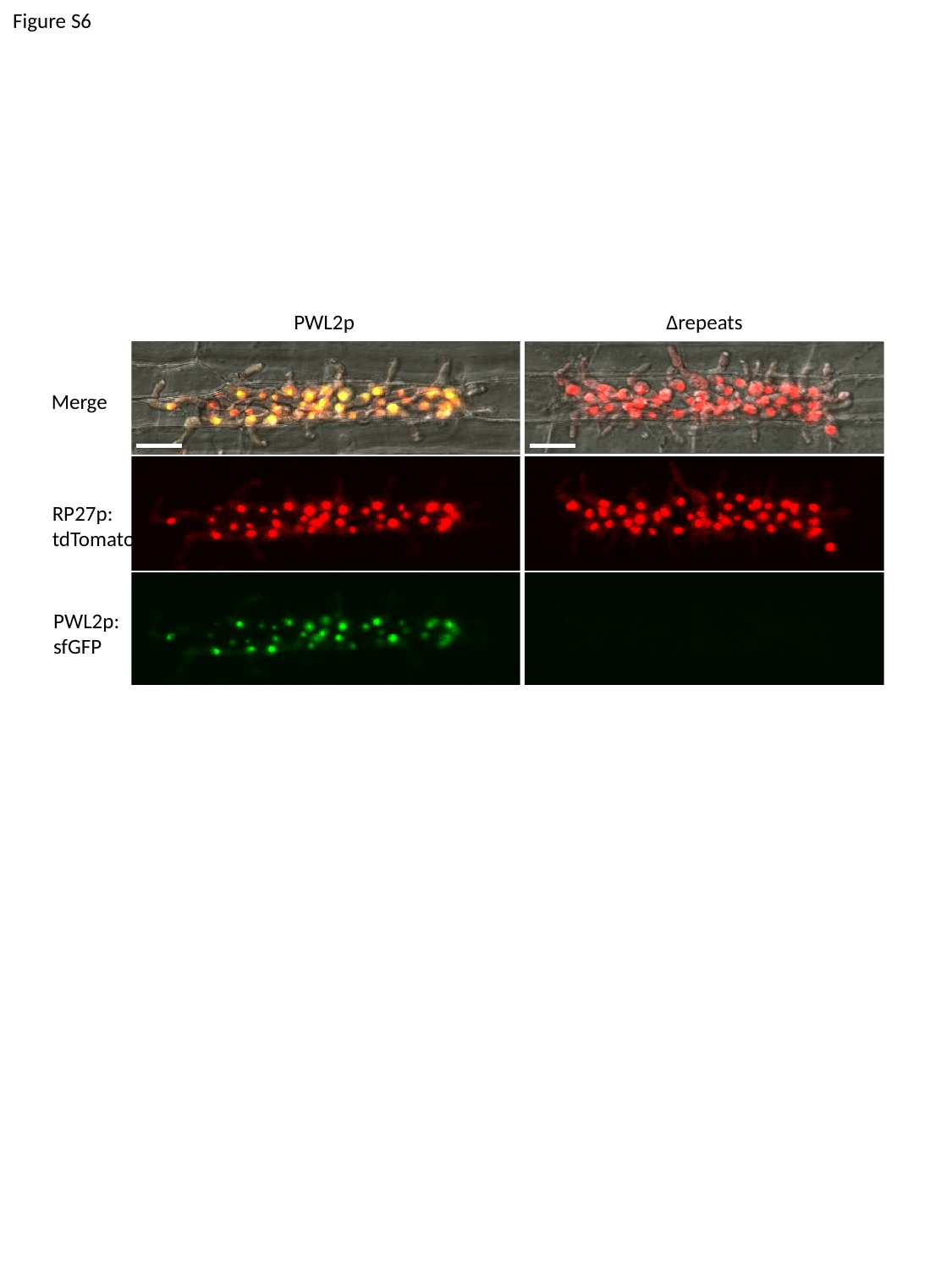

Figure S6
PWL2p
Δrepeats
Merge
RP27p:
tdTomato
PWL2p:
sfGFP

Supplement: Supplementary file 6 — FIGURE S6 The tandem repeats are required for PWL2 induction during cell‐to‐cell movement of invasive hyphae [file MPP-22-508-s002.pptx]

## Slide 1
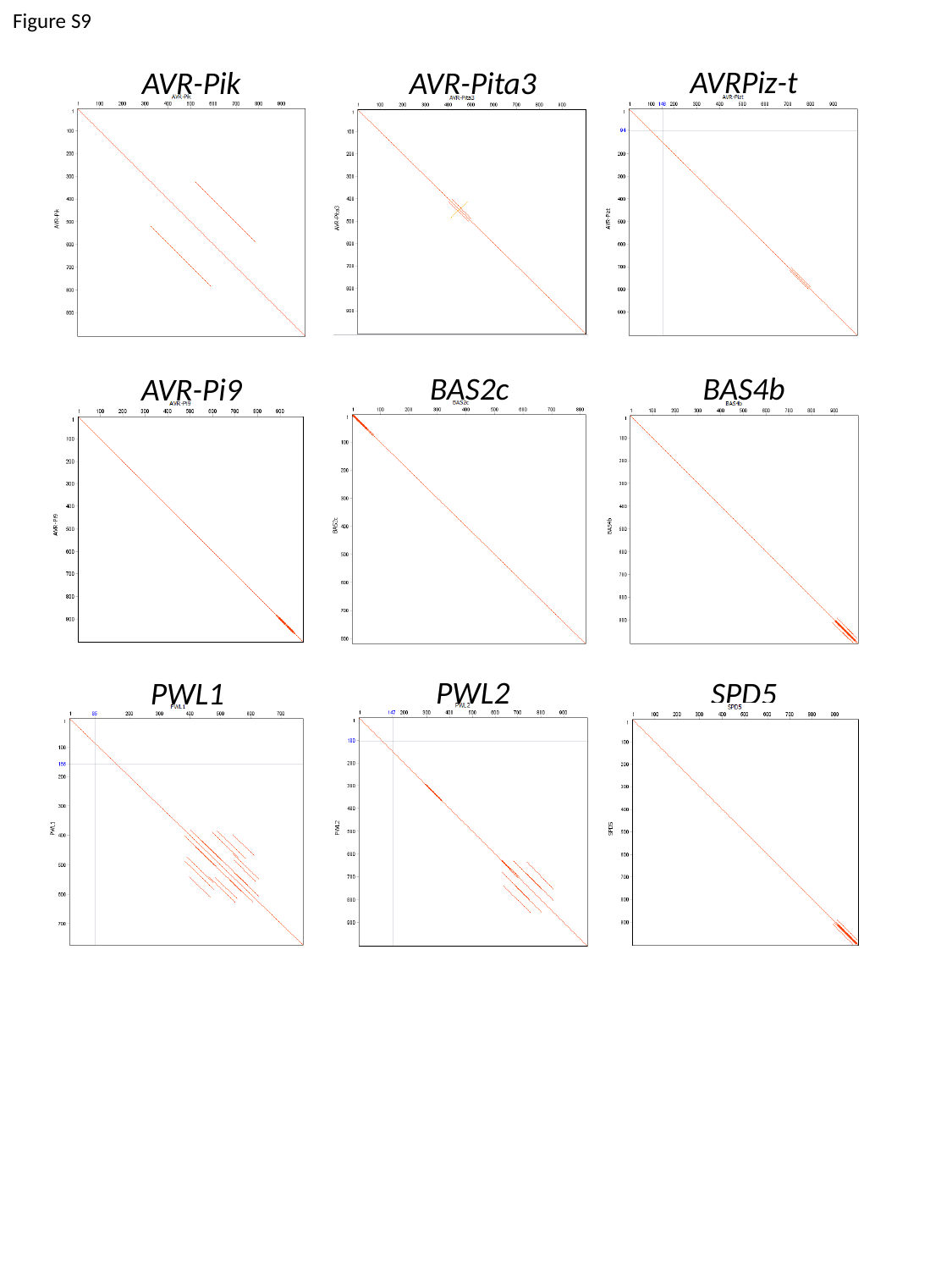

Figure S9
AVRPiz-t
AVR-Pik
AVR-Pita3
BAS2c
BAS4b
AVR-Pi9
PWL2
PWL1
SPD5

Supplement: Supplementary file 9 — FIGURE S9 The promoter regions of multiple effector genes in Magnaporthe oryzae have repeat sequences [file MPP-22-508-s010.pptx]
