# Supplementary material for: Tandem DNA repeats contain cis‐regulatory sequences that activate biotrophy‐specific expression of Magnaporthe effector gene PWL2
Source: Mol Plant Pathol. 2021 Mar 10;22(5):508–21. doi: 10.1111/mpp.13038 (PMC8035637; doi:10.1111/mpp.13038)
Supplement: Supplementary file 3 — FIGURE S3 Confocal images of Magnaporthe oryzae transformant CKF3538 invading rice cells and nonhost onion cells with a highly sensitive setting [file MPP-22-508-s006.pptx]

## Slide 1
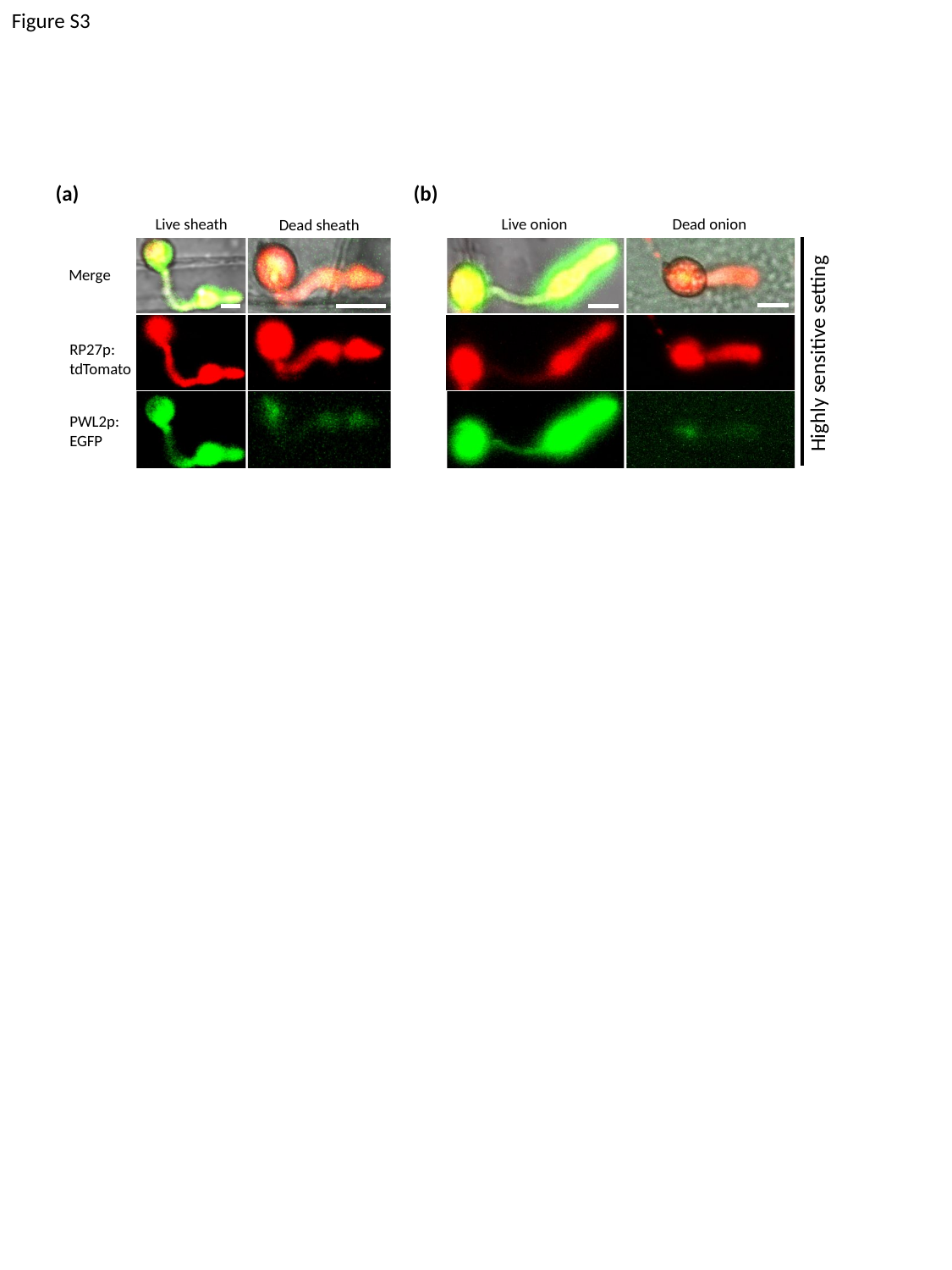

Figure S3
(a)
(b)
Live sheath
Dead onion
Live onion
Dead sheath
Merge
RP27p:
tdTomato
Highly sensitive setting
PWL2p:
EGFP
