# Supplementary material for: Tandem DNA repeats contain cis‐regulatory sequences that activate biotrophy‐specific expression of Magnaporthe effector gene PWL2
Source: Mol Plant Pathol. 2021 Mar 10;22(5):508–21. doi: 10.1111/mpp.13038 (PMC8035637; doi:10.1111/mpp.13038)
Supplement: Supplementary file 7 — FIGURE S7 Graphic presentation of the PWL2 promoter regions and modified tandem repeats, and the sfGFP fusion constructs used in this study [file MPP-22-508-s013.pptx]

## Slide 1
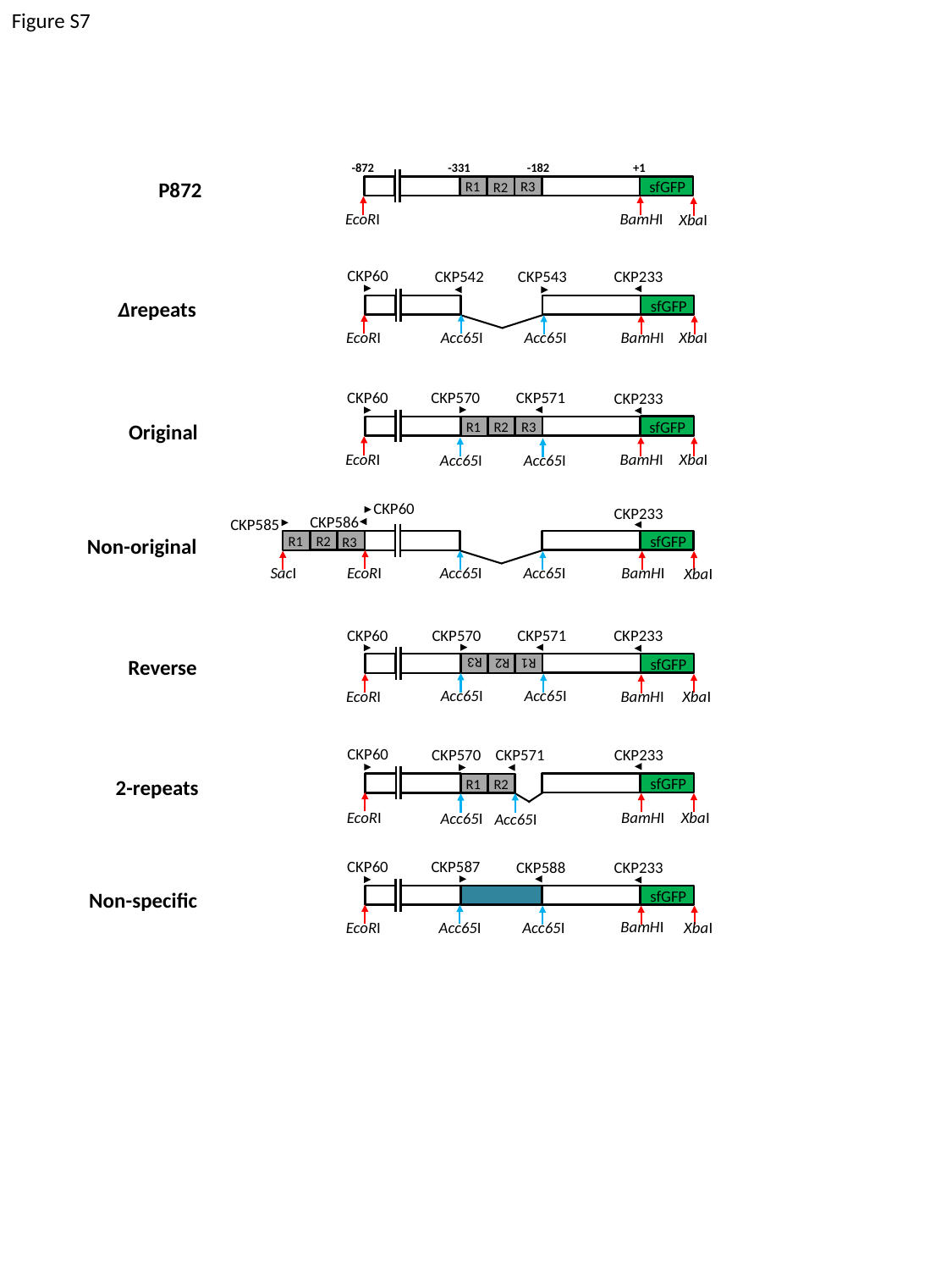

Figure S7
-872
-331
+1
-182
sfGFP
P872
R1
R3
R2
EcoRI
BamHI
XbaI
CKP60
CKP543
CKP233
CKP542
Δrepeats
sfGFP
EcoRI
BamHI
Acc65I
Acc65I
XbaI
CKP60
CKP570
CKP571
CKP233
sfGFP
R1
R3
R2
Original
EcoRI
BamHI
XbaI
Acc65I
Acc65I
CKP60
CKP233
CKP586
CKP585
sfGFP
R1
R2
R3
Non-original
Acc65I
Acc65I
EcoRI
BamHI
SacI
XbaI
CKP60
CKP570
CKP571
CKP233
sfGFP
Reverse
R3
R2
R1
Acc65I
Acc65I
EcoRI
BamHI
XbaI
CKP60
CKP571
CKP233
CKP570
sfGFP
2-repeats
R1
R2
XbaI
BamHI
EcoRI
Acc65I
Acc65I
CKP587
CKP60
CKP588
CKP233
sfGFP
Non-specific
BamHI
XbaI
EcoRI
Acc65I
Acc65I
