# Supplementary material for: Tandem DNA repeats contain cis‐regulatory sequences that activate biotrophy‐specific expression of Magnaporthe effector gene PWL2
Source: Mol Plant Pathol. 2021 Mar 10;22(5):508–21. doi: 10.1111/mpp.13038 (PMC8035637; doi:10.1111/mpp.13038)
Supplement: Supplementary file 8 — FIGURE S8 Graphic presentation of the deletions and substitutions of one repeat, and the sfGFP fusion constructs used in this study [file MPP-22-508-s005.pptx]

## Slide 1
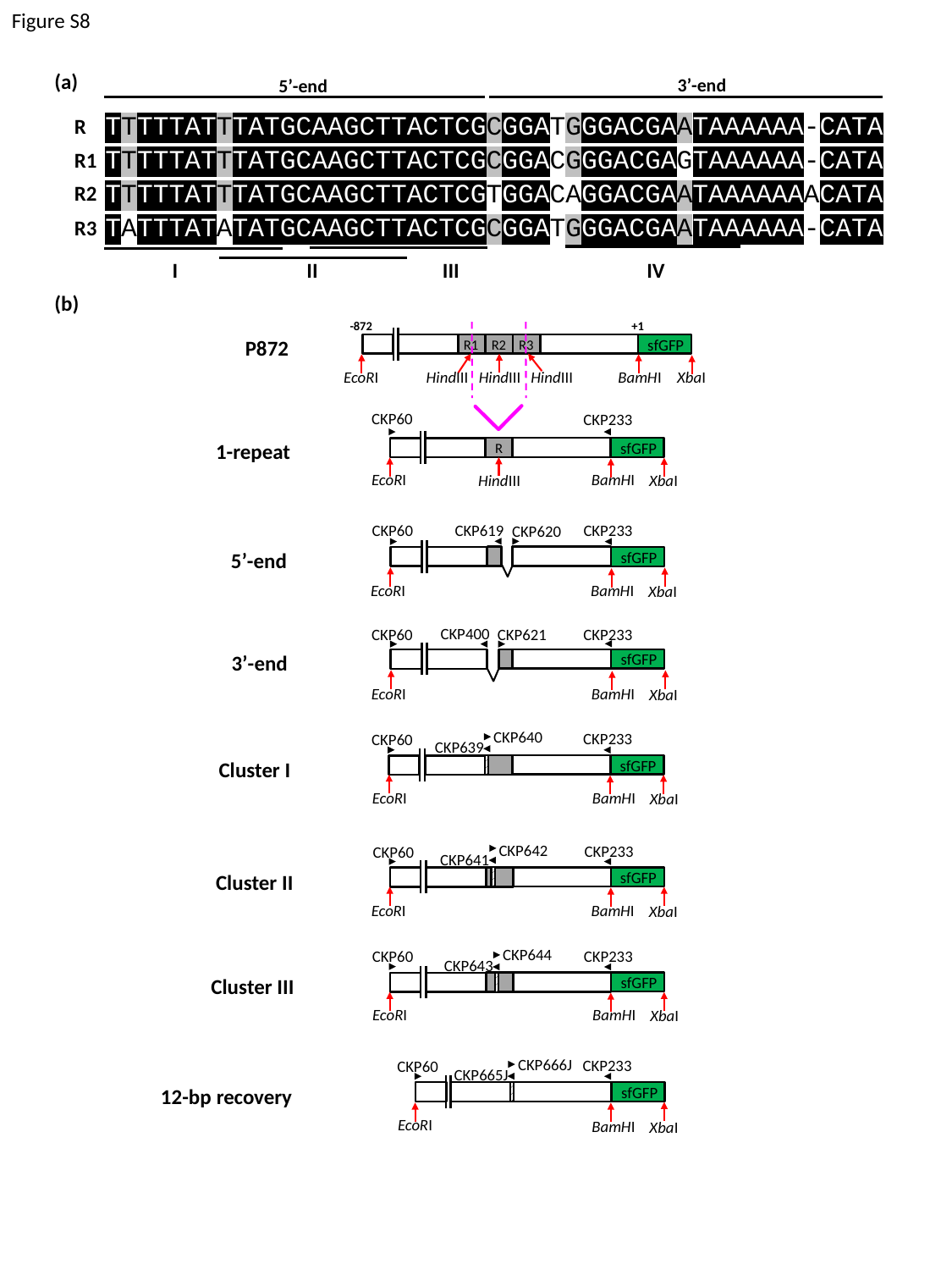

Figure S8
(a)
3’-end
5’-end
TTTTTATTTATGCAAGCTTACTCGCGGATGGGACGAATAAAAAA-CATA
TTTTTATTTATGCAAGCTTACTCGCGGACGGGACGAGTAAAAAA-CATA
TTTTTATTTATGCAAGCTTACTCGTGGACAGGACGAATAAAAAAACATA
TATTTATATATGCAAGCTTACTCGCGGATGGGACGAATAAAAAA-CATA
R
R1
R2
R3
IV
I
III
II
(b)
-872
+1
sfGFP
P872
R1
R3
R2
HindIII
EcoRI
BamHI
HindIII
HindIII
XbaI
CKP60
CKP233
1-repeat
sfGFP
R
EcoRI
BamHI
HindIII
XbaI
CKP233
CKP619
CKP60
CKP620
sfGFP
5’-end
EcoRI
BamHI
XbaI
CKP400
CKP233
CKP60
CKP621
3’-end
sfGFP
EcoRI
BamHI
XbaI
CKP640
CKP233
CKP60
CKP639
sfGFP
Cluster I
EcoRI
BamHI
XbaI
CKP642
CKP233
CKP60
CKP641
sfGFP
Cluster II
EcoRI
BamHI
XbaI
CKP644
CKP233
CKP60
CKP643
sfGFP
Cluster III
EcoRI
BamHI
XbaI
CKP666J
CKP233
CKP60
CKP665J
sfGFP
12-bp recovery
EcoRI
BamHI
XbaI
