# Supplementary material for: Tandem DNA repeats contain cis‐regulatory sequences that activate biotrophy‐specific expression of Magnaporthe effector gene PWL2
Source: Mol Plant Pathol. 2021 Mar 10;22(5):508–21. doi: 10.1111/mpp.13038 (PMC8035637; doi:10.1111/mpp.13038)
Supplement: Supplementary file 10 — TABLE S1 Effector and effector candidate genes identified by BLAST search of the tandem repeat R1 in promoters of Magnaporthe oryzae genes [file MPP-22-508-s003.docx]

**Table S1** Effector and effector candidate genes identified by BLAST search of the tandem repeat R1 in promoters of *M. oryzae* genes.

| Gene | MGG_# | Length(aa) | SP* | Chromosome | Reference |
| --- | --- | --- | --- | --- | --- |
| *BAS1* | MGG_04795 | 115 | Y | IV (I from NCBI) | Mosquera *et al.*, 2009 |
| *BAS2* | MGG_09693 | 102 | Y | V(4 from NCBI) | Mosquera *et al.*, 2009 |
| *MAX* | MGG_08482 | 142 | Y | 4 | Guillen *et al.*, 2015 |
| *AVR-Pita family, metalloproteinase* | MGG_14981 | 226 | Y | UK | Khang *et al.*, 2008 |
| *Candidate effector* | MGG_14156 | 156 | Y | 6 | Dong *et al.*, 2015 |
| *Candidate effector* | MGG_18108 | 174 | Y | UK | Dong *et al.*, 2015 |
| *Candidate effector* | MGG_18105 | 123 | Y | UK | Dong *et al.*, 2015 |
| *Candidate effector* | MGG_17244 | 74 | Y | 4 | Dong *et al.*, 2015 |
| *Candidate effector* | MGG_01953 | 115 | Y | 6 | Dong *et al.*, 2015 |
| *Candidate effector* | MGG_09019 | 193 | Y | 7 | Dong *et al.*, 2015 |
| *Candidate effector* | MGG_08715 | 137 | Y | 6 | Dong *et al.*, 2015 |
| *Candidate effector* | MGG_08799 | 116 | Y | 6 | Dong *et al.*, 2015 |
| *Candidate effector* | MGG_09605 | 182 | Y | 7 | Dong *et al.*, 2015 |
| *Candidate effector* | MGG_17239 | 70 | Y | 4 | Dong *et al.*, 2015 |
| *Candidate effector* | MGG_17425 | 86 | Y | 5 | Dong *et al.*, 2015 |

*SP indicates signal peptide that is predicted by SignalP 4.0 Server.
