# Supplementary material for: Tandem DNA repeats contain cis‐regulatory sequences that activate biotrophy‐specific expression of Magnaporthe effector gene PWL2
Source: Mol Plant Pathol. 2021 Mar 10;22(5):508–21. doi: 10.1111/mpp.13038 (PMC8035637; doi:10.1111/mpp.13038)
Supplement: Supplementary file 11 — TABLE S2 Predicted effector genes with a 12‐bp‐like motif in promoters [file MPP-22-508-s004.docx]

**Table S2** Predicted effector genes with a 12-bp like motif in promoters.

| **#** | **MGG_#** | **Description** | **Strand** | **Start** | **End** | **p-value** | **q-value** | **Matched Sequence** |
| --- | --- | --- | --- | --- | --- | --- | --- | --- |
| 1 | MGG_00043 | MoHEG9 | - | 439 | 450 | 8.27E-05 | 0.785 | GTATGCAAGCCG |
| 2 | MGG_00043 | hypothetical protein | + | 223 | 234 | 2.84E-05 | 0.652 | TAATGCAACCTT |
| 3 | MGG_00052 | hypothetical protein | + | 427 | 438 | 9.30E-05 | 0.818 | TTGTGCCAGCGT |
| 4 | MGG_00225 | hypothetical protein | - | 68 | 79 | 5.20E-06 | 0.368 | AGATGCAAGCTT |
| 5 | MGG_00230 | hypothetical protein | + | 209 | 220 | 9.69E-05 | 0.818 | GGATGCATGCTT |
| 6 | MGG_00269 | hypothetical protein | - | 77 | 88 | 9.69E-05 | 0.818 | TGTGGCAAGCTT |
| 7 | MGG_00269 | hypothetical protein | + | 275 | 286 | 3.87E-05 | 0.654 | TTACCCAAGCTT |
| 8 | MGG_01145 | hypothetical protein | + | 790 | 801 | 5.23E-05 | 0.773 | GTAAGCAAGCTA |
| 9 | MGG_01173 | hydrophobin | + | 905 | 916 | 1.46E-05 | 0.652 | TTTTGCAAGCAT |
| 10 | MGG_01366 | hypothetical protein | - | 569 | 580 | 3.49E-05 | 0.654 | TTTTGCAAACTT |
| 11 | MGG_01530 | hypothetical protein | - | 688 | 699 | 6.21E-05 | 0.785 | GTAGGCGAGCTT |
| 12 | MGG_01900 | hypothetical protein | - | 79 | 90 | 4.35E-05 | 0.701 | TTTTGCAATCTT |
| 13 | MGG_01953 | hypothetical protein | + | 692 | 703 | 8.43E-07 | 0.0897 | TTATGCAAGCTG |
| 14 | MGG_01964 | hypothetical protein | + | 561 | 572 | 2.84E-05 | 0.652 | TAATGCAACCTT |
| 15 | MGG_02212 | hypothetical protein | - | 861 | 872 | 4.08E-05 | 0.668 | TTATGCAATCCT |
| 16 | MGG_02220 | hypothetical protein | - | 164 | 175 | 4.74E-05 | 0.741 | TTATTCAAGATT |
| 17 | MGG_02220 | hypothetical protein | + | 968 | 979 | 4.87E-05 | 0.751 | TTATCCAATCTT |
| 18 | MGG_02273 | hypothetical protein | + | 561 | 572 | 7.71E-05 | 0.785 | GTCTGCAAGCTA |
| 19 | MGG_02338 | hypothetical protein | - | 733 | 744 | 2.38E-05 | 0.652 | TTAGACAAGCTT |
| 20 | MGG_02590 | hypothetical protein | + | 80 | 91 | 8.43E-07 | 0.0897 | GTATGCAAGCTT |
| 21 | MGG_02645 | hypothetical protein | - | 325 | 336 | 6.70E-05 | 0.785 | TACTGCAAGCTA |
| 22 | MGG_03308 | hypothetical protein | + | 223 | 234 | 6.22E-06 | 0.389 | ATGTGCAAGCTT |
| 23 | MGG_03308 | hypothetical protein | + | 554 | 565 | 8.68E-05 | 0.81 | TTCTGCAAGCGG |
| 24 | MGG_03308 | hypothetical protein | + | 578 | 589 | 6.21E-05 | 0.785 | GTATGCGAGCTG |
| 25 | MGG_03338 | cellulose-binding protein | + | 598 | 609 | 3.37E-05 | 0.654 | TTATGGCAGCTT |
| 26 | MGG_03495 | hypothetical protein | - | 687 | 698 | 7.03E-05 | 0.785 | TGAAGCAAGCCT |
| 27 | MGG_03495 | hypothetical protein | + | 297 | 308 | 1.60E-05 | 0.652 | TTTTGCGAGCTT |
| 28 | MGG_03507 | hypothetical protein | + | 351 | 362 | 9.52E-05 | 0.818 | TTTTGCAGGCTA |
| 29 | MGG_03639 | hypothetical protein | - | 716 | 727 | 3.65E-05 | 0.654 | GTATGCAAGTTT |
| 30 | MGG_04301 | PWL2 | + | 677 | 688 | 8.34E-08 | 0.0222 | TTATGCAAGCTT |
| 31 | MGG_04301 | PWL2 | + | 727 | 738 | 8.34E-08 | 0.0222 | TTATGCAAGCTT |
| 32 | MGG_04301 | PWL2 | + | 779 | 790 | 5.00E-07 | 0.0665 | ATATGCAAGCTT |
| 33 | MGG_04451 | hypothetical protein | - | 396 | 407 | 9.30E-05 | 0.818 | TGTAGCAAGCTT |
| 34 | MGG_04451 | hypothetical protein | - | 759 | 770 | 5.20E-06 | 0.368 | TGATGCAAGCTA |
| 35 | MGG_04507 | synbindin | + | 918 | 929 | 9.69E-05 | 0.818 | GGTTGCAAGCTT |
| 36 | MGG_04859 | hypothetical protein | - | 27 | 38 | 5.03E-05 | 0.753 | AAAAGCAAGCTT |
| 37 | MGG_05091 | hypothetical protein | - | 154 | 165 | 8.27E-05 | 0.785 | GCATGCAAGCGT |
| 38 | MGG_05608 | hypothetical protein | - | 915 | 926 | 8.27E-05 | 0.785 | AAATGCATGCTT |
| 39 | MGG_05818 | hypothetical protein | - | 680 | 691 | 1.56E-05 | 0.652 | TTATGCATGCGT |
| 40 | MGG_05831 | hypothetical protein | - | 232 | 243 | 1.86E-05 | 0.652 | TCTTGCAAGCTT |
| 41 | MGG_05896 | hypothetical protein | + | 272 | 283 | 7.71E-05 | 0.785 | TAAGGCACGCTT |
| 42 | MGG_06008 | hypothetical protein | - | 861 | 872 | 6.07E-05 | 0.785 | TAGTGCAGGCTT |
| 43 | MGG_06359 | hypothetical protein | + | 331 | 342 | 9.52E-05 | 0.818 | TAGTGCTAGCTT |
| 44 | MGG_07234 | FK506-binding protein 2 | + | 973 | 984 | 5.00E-07 | 0.0665 | TAATGCAAGCTT |
| 45 | MGG_07538 | hypothetical protein | - | 40 | 51 | 8.27E-05 | 0.785 | TTCAGCAGGCTT |
| 46 | MGG_07538 | hypothetical protein | + | 863 | 874 | 3.80E-05 | 0.654 | TTATGGTAGCTT |
| 47 | MGG_07607 | hypothetical protein | - | 843 | 854 | 6.28E-05 | 0.785 | TTAGGCGGGCTT |
| 48 | MGG_07810 | hypothetical protein | + | 486 | 497 | 7.71E-05 | 0.785 | TAAGGCACGCTT |
| 49 | MGG_07919 | hypothetical protein | - | 20 | 31 | 8.27E-05 | 0.785 | TTTTGCAAGCAA |
| 50 | MGG_07952 | hypothetical protein | - | 638 | 649 | 3.49E-05 | 0.654 | TTATGCTAACTT |
| 51 | MGG_07986 | MoCDIP3 | - | 929 | 940 | 7.71E-05 | 0.785 | TTCTGCAAGCGA |
| 52 | MGG_08027 | hypothetical protein | - | 222 | 233 | 7.71E-05 | 0.785 | TACGGCAAGCTT |
| 53 | MGG_08300 | hypothetical protein | + | 789 | 800 | 8.27E-05 | 0.785 | GCATGCAAGCTG |
| 54 | MGG_08407 | hypothetical protein | - | 964 | 975 | 9.30E-05 | 0.818 | TGCTGCAGGCTT |
| 55 | MGG_08407 | hypothetical protein | + | 430 | 441 | 6.49E-05 | 0.785 | AAATGCAAGCCT |
| 56 | MGG_08414 | Max | - | 453 | 464 | 3.87E-05 | 0.654 | TTACGCAACCTT |
| 57 | MGG_08451 | hypothetical protein | - | 5 | 16 | 4.74E-05 | 0.741 | TTATTCAAGATT |
| 58 | MGG_08451 | hypothetical protein | - | 92 | 103 | 6.49E-05 | 0.785 | CAAAGCAAGCTT |
| 59 | MGG_08451 | hypothetical protein | - | 957 | 968 | 5.94E-05 | 0.785 | TTGAGCAAGCTG |
| 60 | MGG_08451 | hypothetical protein | + | 86 | 97 | 9.30E-05 | 0.818 | AGTTGCAAGCTT |
| 61 | MGG_08609 | hypothetical protein | + | 770 | 781 | 7.71E-05 | 0.785 | TCATGCGAGCTA |
| 62 | MGG_08799 | hypothetical protein | - | 599 | 610 | 7.03E-05 | 0.785 | CAATGCAAGCTG |
| 63 | MGG_08817 | hypothetical protein | - | 658 | 669 | 7.03E-05 | 0.785 | CTATGCAAGCGA |
| 64 | MGG_08941 | hypothetical protein | + | 365 | 376 | 9.30E-05 | 0.818 | TGTTGCAAGCAT |
| 65 | MGG_09425 | Max | - | 567 | 578 | 9.52E-05 | 0.818 | TTGTGCATGCAT |
| 66 | MGG_09724 | hypothetical protein | - | 770 | 781 | 4.08E-05 | 0.668 | TCATTCAAGCTT |
| 67 | MGG_09842 | hypothetical protein | - | 925 | 936 | 2.59E-05 | 0.652 | TTGTGCAAGGTT |
| 68 | MGG_09844 | hypothetical protein | + | 559 | 570 | 7.63E-06 | 0.451 | TTGTGCAAGCGT |
| 69 | MGG_09998 | hypothetical protein | - | 359 | 370 | 8.27E-05 | 0.785 | TAATGCATGCTA |
| 70 | MGG_10026 | cysteine rich protein | + | 791 | 802 | 2.94E-05 | 0.652 | TTATGACAGCTT |
| 71 | MGG_10217 | hypothetical protein | + | 60 | 71 | 3.65E-05 | 0.654 | TGATTCAAGCTT |
| 72 | MGG_10259 | hypothetical protein | - | 488 | 499 | 7.03E-05 | 0.785 | GTATGCAAGCCA |
| 73 | MGG_10276 | hypothetical protein | - | 515 | 526 | 8.27E-05 | 0.785 | ATATGCTAGCAT |
| 74 | MGG_10276 | hypothetical protein | + | 513 | 524 | 8.27E-05 | 0.785 | TAATGCTAGCAT |
| 75 | MGG_10456 | hypothetical protein | + | 672 | 683 | 2.94E-05 | 0.652 | TTATGCCAGATT |
| 76 | MGG_10914 | BAS4 | - | 941 | 952 | 2.84E-05 | 0.652 | ATATCCAAGCTT |
| 77 | MGG_11650 | hypothetical protein | + | 735 | 746 | 8.68E-05 | 0.81 | TGGTGCAAGCTC |
| 78 | MGG_11967 | hypothetical protein | - | 305 | 316 | 3.49E-05 | 0.654 | TTTTACAAGCTT |
| 79 | MGG_11991 | SPD10 | + | 105 | 116 | 1.56E-05 | 0.652 | TGATGCATGCTT |
| 80 | MGG_12415 | hypothetical protein | + | 685 | 696 | 1.39E-06 | 0.134 | TTACGCAAGCTT |
| 81 | MGG_12445 | hypothetical protein | + | 908 | 919 | 1.60E-05 | 0.652 | TTTTGCAGGCTT |
| 82 | MGG_12466 | hypothetical protein | + | 983 | 994 | 7.71E-05 | 0.785 | TGAAGCCAGCTT |
| 83 | MGG_12552 | hypothetical protein | + | 829 | 840 | 2.84E-05 | 0.652 | TCATACAAGCTT |
| 84 | MGG_12654 | hypothetical protein | + | 802 | 813 | 7.71E-05 | 0.785 | TTCTGCAAGCAG |
| 85 | MGG_13019 | hypothetical protein | + | 40 | 51 | 3.37E-05 | 0.654 | TTATTCAAGCAT |
| 86 | MGG_13863 | PWL2 | + | 677 | 688 | 8.34E-08 | 0.0222 | TTATGCAAGCTT |
| 87 | MGG_13863 | PWL2 | + | 727 | 738 | 8.34E-08 | 0.0222 | TTATGCAAGCTT |
| 88 | MGG_13863 | PWL2 | + | 779 | 790 | 5.00E-07 | 0.0665 | ATATGCAAGCTT |
| 89 | MGG_13868 | hypothetical protein | + | 486 | 497 | 7.71E-05 | 0.785 | TAAGGCACGCTT |
| 90 | MGG_14006 | hypothetical protein | - | 580 | 591 | 1.13E-05 | 0.634 | TGATGCAAGCTC |
| 91 | MGG_14374 | hypothetical protein | - | 596 | 607 | 1.76E-05 | 0.652 | TTATGCCCGCTT |
| 92 | MGG_14652 | hypothetical protein | + | 97 | 108 | 1.30E-05 | 0.652 | TTGTGCAAGCTC |
| 93 | MGG_14836 | hypothetical protein | - | 232 | 243 | 5.00E-07 | 0.0665 | ATATGCAAGCTT |
| 94 | MGG_15106 | hypothetical protein | + | 885 | 896 | 7.71E-05 | 0.785 | TAAGGCCAGCTT |
| 95 | MGG_15374 | hypothetical protein | + | 332 | 343 | 8.27E-05 | 0.785 | ATAAGCATGCTT |
| 96 | MGG_15410 | hypothetical protein | - | 121 | 132 | 7.71E-05 | 0.785 | GTAAGCACGCTT |
| 97 | MGG_15539 | hypothetical protein | - | 738 | 749 | 3.49E-05 | 0.654 | TTTTGCAAGATT |
| 98 | MGG_15703 | hypothetical protein | + | 69 | 80 | 6.21E-05 | 0.785 | GGATGCGAGCTT |
| 99 | MGG_15793 | hypothetical protein | - | 489 | 500 | 3.37E-05 | 0.654 | ATATGCAAGTTT |
| 100 | MGG_15972 | Avr-Pik | + | 468 | 479 | 3.83E-06 | 0.313 | TAATGCAAGCAT |
| 101 | MGG_15972 | Avr-Pik | + | 665 | 676 | 3.83E-06 | 0.313 | TAATGCAAGCAT |
| 102 | MGG_15973 | hypothetical protein | - | 561 | 572 | 3.80E-05 | 0.654 | TTTTGGAAGCTT |
| 103 | MGG_16041 | hypothetical protein | - | 856 | 867 | 7.71E-05 | 0.785 | TTCTGCAAGCGA |
| 104 | MGG_16058 | hypothetical protein | + | 765 | 776 | 2.59E-05 | 0.652 | TTGTGCAAGGTT |
| 105 | MGG_16059 | hypothetical protein | - | 814 | 825 | 1.74E-05 | 0.652 | CTATGCCAGCTT |
| 106 | MGG_16175 | hypothetical protein | - | 490 | 501 | 2.84E-05 | 0.652 | TTATGCAAACTC |
| 107 | MGG_16188 | hypothetical protein | - | 650 | 661 | 7.71E-05 | 0.785 | TTATGCAGGCAC |
| 108 | MGG_16345 | hypothetical protein | + | 674 | 685 | 2.38E-05 | 0.652 | TGATGAAAGCTT |
| 109 | MGG_16357 | hypothetical protein | + | 917 | 928 | 9.30E-05 | 0.818 | TTTTGCAAGCAG |
| 110 | MGG_16553 | hypothetical protein | - | 494 | 505 | 3.37E-05 | 0.654 | ATATGCAAGTTT |
| 111 | MGG_16698 | hypothetical protein | + | 614 | 625 | 2.84E-05 | 0.652 | TAATGCAACCTT |
| 112 | MGG_16737 | hypothetical protein | + | 318 | 329 | 5.61E-05 | 0.785 | GTATGCAAGCAG |
| 113 | MGG_16939 | hypothetical protein | - | 364 | 375 | 2.13E-05 | 0.652 | TAATGCAAACTT |
| 114 | MGG_17022 | hypothetical protein | - | 73 | 84 | 1.93E-05 | 0.652 | TTTTGCTAGCTT |
| 115 | MGG_17425 | hypothetical protein | - | 241 | 252 | 3.87E-05 | 0.654 | TTATCCAAGCTC |
| 116 | MGG_17463 | hypothetical protein | - | 766 | 777 | 8.27E-05 | 0.785 | TCATGCAAGCGG |
| 117 | MGG_17567 | hypothetical protein | - | 293 | 304 | 2.84E-05 | 0.652 | TTACACAAGCTT |
| 118 | MGG_17567 | hypothetical protein | + | 53 | 64 | 5.03E-05 | 0.753 | AAAAGCAAGCTT |
| 119 | MGG_17579 | hypothetical protein | + | 465 | 476 | 2.59E-05 | 0.652 | TTGTGCAAGGTT |
| 120 | MGG_17614 | hypothetical protein | + | 481 | 492 | 2.84E-05 | 0.652 | TAATGCAACCTT |
| 121 | MGG_17635 | hypothetical protein | + | 850 | 861 | 2.13E-05 | 0.652 | TAATGCAAACTT |
| 122 | MGG_17711 | hypothetical protein | + | 377 | 388 | 6.22E-06 | 0.389 | ATATGCAGGCTT |
| 123 | MGG_18013 | hypothetical protein | - | 985 | 996 | 6.28E-05 | 0.785 | TTGGGCGAGCTT |
| 124 | MGG_18013 | hypothetical protein | + | 348 | 359 | 2.13E-05 | 0.652 | ATATGAAAGCTT |
| 125 | MGG_18062 | hypothetical protein | - | 365 | 376 | 2.13E-05 | 0.652 | TAATGCAAACTT |
| 126 | MGG_18122 | hypothetical protein | - | 493 | 504 | 7.03E-05 | 0.785 | GTACGCAAGCAT |
