# Supplementary material for: Tandem DNA repeats contain cis‐regulatory sequences that activate biotrophy‐specific expression of Magnaporthe effector gene PWL2
Source: Mol Plant Pathol. 2021 Mar 10;22(5):508–21. doi: 10.1111/mpp.13038 (PMC8035637; doi:10.1111/mpp.13038)
Supplement: Supplementary file 12 — TABLE S3 Magnaporthe oryzae strains used in this study [file MPP-22-508-s009.docx]

**Table S3** *Magnaporthe oryzae* strains used in this study.

| Strain | CKF# | Genotype | Reference |
| --- | --- | --- | --- |
| O-137 | CKF558 | Wild-type, a field isolate from rice in China. | Sweigard *et al.*, 1995 |
| Recipient strain | CKF3209 | Transformant of CKF558, expressing a nuclear tdTomato reporter gene under control of a constitutive promoter *M. oryzae* *RP27*; G418^R^ | This study |
| PWL2 reporter strain(nuclear) | CKF3276 | Transformant of CKF3209, expressing both a constitutive, nuclear tdTomato reporter gene, and a fusion of the *PWL2* promoter with sfGFP:NLS reporter gene; Hyg^R^ G418^R^ | This study |
| PWL2 reporter strain(nuclear) | CKF3278 | Transformant of CKF3209, expressing both a constitutive, nuclear tdTomato reporter gene, and a fusion of the *PWL2* promoter with sfGFP:NLS reporter gene; Hyg^R^ G418^R^ | This study |
| PWL2 reporter strain (cytoplasmic) | CKF3538 | Transformant of CKF558, expressing both a constitutive, cytoplasmic tdTomato reporter gene, and a fusion of the *PWL2* promoter with EGFP:PEST reporter gene; Hyg^R^ G418^R^ | This study |
| *Δ*repeats | CKF3692 | Transformant of CKF3209, expressing both a constitutive, nuclear tdTomato reporter gene, and a fusion of the *PWL2* promoter (without 3-repeats) with sfGFP:NLS reporter gene; Hyg^R^ G418^R^ | This study |
| *Δ*repeats | CKF3700 | Transformant of CKF3209, expressing both a constitutive, nuclear tdTomato reporter gene, and a fusion of the *PWL2* promoter (without 3-repeats) with sfGFP:NLS reporter gene; Hyg^R^ G418^R^ | This study |
| 1-repeat | CKF3736 | Transformant of CKF3209, expressing both a constitutive, nuclear tdTomato reporter gene, and a fusion of the *PWL2* promoter (1-repeat) with sfGFP:NLS reporter gene; Hyg^R^ G418^R^ | This study |
| 1-repeat | CKF3737 | Transformant of CKF3209, expressing both a constitutive, nuclear tdTomato reporter gene, and a fusion of the *PWL2* promoter (1-repeat) with sfGFP:NLS reporter gene; Hyg^R^ G418^R^ | This study |
| Reversed orientation of 3-repeats | CKF3745 | Transformant of CKF3209, expressing both a constitutive, nuclear tdTomato reporter gene, and a fusion of the *PWL2* promoter (reversed repeats) with sfGFP:NLS reporter gene; HygR G418^R^ | This study |
| Reversed orientation of 3-repeats | CKF3748 | Transformant of CKF3209, expressing both a constitutive, nuclear tdTomato reporter gene, and a fusion of the *PWL2* promoter (reversed repeats) with sfGFP:NLS reporter gene; Hyg^R^ G418^R^ | This study |
| Reversed orientation of 3-repeats | CKF3751 | Transformant of CKF3209, expressing both a constitutive, nuclear tdTomato reporter gene, and a fusion of the *PWL2* promoter (reversed repeats) with sfGFP:NLS reporter gene; Hyg^R^ G418^R^ | This study |
| Original position of 3-repeats | CKF3757 | Transformant of CKF3209, expressing both a constitutive, nuclear tdTomato reporter gene, and a fusion of the *PWL2* promoter (forward repeats) with sfGFP:NLS reporter gene; Hyg^R^ G418^R^ | This study |
| Original position of 3-repeats | CKF3758 | Transformant of CKF3209, expressing both a constitutive, nuclear tdTomato reporter gene, and a fusion of the *PWL2* promoter (forward repeats) with sfGFP:NLS reporter gene; Hyg^R^ G418^R^ | This study |
| Original position of 3-repeats | CKF3760 | Transformant of CKF3209, expressing both a constitutive, nuclear tdTomato reporter gene, and a fusion of the *PWL2* promoter (forward repeats) with sfGFP:NLS reporter gene; Hyg^R^ G418^R^ | This study |
| Repeats at non-original position | CKF3778 | Transformant of CKF3209, expressing both a constitutive, nuclear tdTomato reporter gene, and a fusion of the *PWL2* promoter (repeats at non-original position) with sfGFP:NLS reporter gene; Hyg^R^ G418^R^ | This study |
| Repeats at non-original position | CKF3780 | Transformant of CKF3209, expressing both a constitutive, nuclear tdTomato reporter gene, and a fusion of the *PWL2* promoter (repeats at non-original position) with sfGFP:NLS reporter gene; Hyg^R^ G418^R^ | This study |
| 2-repeats | CKF3782 | Transformant of CKF3209, expressing both a constitutive, nuclear tdTomato reporter gene, and a fusion of the *PWL2* promoter (2-repeats) with sfGFP:NLS reporter gene; Hyg^R^ G418^R^ | This study |
| 2-repeats | CKF3784 | Transformant of CKF3209, expressing both a constitutive, nuclear tdTomato reporter gene, and a fusion of the *PWL2* promoter (2-repeats) with sfGFP:NLS reporter gene; HygR G418^R^ | This study |
| 2-repeats | CKF3785 | Transformant of CKF3209, expressing both a constitutive, nuclear tdTomato reporter gene, and a fusion of the *PWL2* promoter (2-repeats) with sfGFP:NLS reporter gene; Hyg^R^ G418^R^ | This study |
| Non-specific DNA replacement | CKF3802 | Transformant of CKF3209, expressing both a constitutive, nuclear tdTomato reporter gene, and a fusion of the *PWL2* promoter (non-specific DNA replacement) with sfGFP:NLS reporter gene; Hyg^R^ G418^R^ | This study |
| Non-specific DNA replacement | CKF3803 | Transformant of CKF3209, expressing both a constitutive, nuclear tdTomato reporter gene, and a fusion of the *PWL2* promoter (non-specific DNA replacement) with sfGFP:NLS reporter gene; Hyg^R^ G418^R^ | This study |
| 5’-end of one repeat | CKF3820 | Transformant of CKF3209, expressing both a constitutive, nuclear tdTomato reporter gene, and a fusion of the *PWL2* promoter (5’-end of one repeat) with sfGFP:NLS reporter gene; Hyg^R^ G418^R^ | This study |
| 5’-end of one repeat | CKF3821 | Transformant of CKF3209, expressing both a constitutive, nuclear tdTomato reporter gene, and a fusion of the *PWL2* promoter (5’-end of one repeat) with sfGFP:NLS reporter gene; HygR G418^R^ | This study |
| 3’-end of one repeat | CKF3852 | Transformant of CKF3209, expressing both a constitutive, nuclear tdTomato reporter gene, and a fusion of the *PWL2* promoter (3’-end of one repeat) with sfGFP:NLS reporter gene; Hyg^R^ G418^R^ | This study |
| 3’-end of one repeat | CKF3853 | Transformant of CKF3209, expressing both a constitutive, nuclear tdTomato reporter gene, and a fusion of the *PWL2* promoter (3’-end of one repeat) with sfGFP:NLS reporter gene; Hyg^R^ G418^R^ | This study |
| Region II mutation | CKF3885 | Transformant of CKF3209, expressing both a constitutive, nuclear tdTomato reporter gene, and a fusion of the *PWL2* promoter (cluster II mutation) with sfGFP:NLS reporter gene; Hyg^R^ G418^R^ | This study |
| Region II mutation | CKF3888 | Transformant of CKF3209, expressing both a constitutive, nuclear tdTomato reporter gene, and a fusion of the *PWL2* promoter (cluster II mutation) with sfGFP:NLS reporter gene; Hyg^R^ G418^R^ | This study |
| Region I mutation | CKF3902 | Transformant of CKF3209, expressing both a constitutive, nuclear tdTomato reporter gene, and a fusion of the *PWL2* promoter (cluster I mutation) with sfGFP:NLS reporter gene; Hyg^R^ G418^R^ | This study |
| Region I mutation | CKF3906 | Transformant of CKF3209, expressing both a constitutive, nuclear tdTomato reporter gene, and a fusion of the *PWL2* promoter (cluster I mutation) with sfGFP:NLS reporter gene; Hyg^R^ G418^R^ | This study |
| Region III mutation | CKF3915 | Transformant of CKF3209, expressing both a constitutive, nuclear tdTomato reporter gene, and a fusion of the *PWL2* promoter (cluster III mutation) with sfGFP:NLS reporter gene; Hyg^R^ G418^R^ | This study |
| Region III mutation | CKF3916 | Transformant of CKF3209, expressing both a constitutive, nuclear tdTomato reporter gene, and a fusion of the *PWL2* promoter (cluster III mutation) with sfGFP:NLS reporter gene; Hyg^R^ G418^R^ | This study |
| 12-bp recovery | CKF3988 | Transformant of CKF3209, expressing both a constitutive, nuclear tdTomato reporter gene, and a fusion of the *PWL2* promoter (12-bp recovery) with sfGFP:NLS reporter gene; Hyg^R^ G418^R^ | This study |
| 12-bp recovery | CKF3989 | Transformant of CKF3209, expressing both a constitutive, nuclear tdTomato reporter gene, and a fusion of the *PWL2* promoter (12-bp recovery) with sfGFP:NLS reporter gene; Hyg^R^ G418^R^ | This study |
| 12-bp recovery | CKF3991 | Transformant of CKF3209, expressing both a constitutive, nuclear tdTomato reporter gene, and a fusion of the *PWL2* promoter (12-bp recovery) with sfGFP:NLS reporter gene; Hyg^R^ G418^R^ | This study |
