# Supplementary material for: Tandem DNA repeats contain cis‐regulatory sequences that activate biotrophy‐specific expression of Magnaporthe effector gene PWL2
Source: Mol Plant Pathol. 2021 Mar 10;22(5):508–21. doi: 10.1111/mpp.13038 (PMC8035637; doi:10.1111/mpp.13038)
Supplement: Supplementary file 13 — TABLE S4 Key plasmids used in this study [file MPP-22-508-s001.docx]

**Table S4** Key plasmids used in this study.

| **Clone** | **Description** |
| --- | --- |
| pBV578 | 0.1-kb nuclear localization signal sequence (NLS, *Bsr*GI*-Bam*HI fragment) and 0.3-kb Nos terminator (*Bgl*II*-Eco*RI fragment) cloned in *BsrG*I*-EcoR*I sites of pAN583 (pBV360, Nelson et al., 2007). |
| pBV1102 | 0.5-kb *PWL2* 3’-UTR (PCR product of pCB775 (Sweigard et al., 1995) with primers CKP267 and CKP268) into pGEM-T (Promega). |
| pCK1292 | Cytoplasmic tdTomato expression binary vector derived from pBV141(pBGt, Kim *et al.*, 2011), consisting of 0.5-kb *RP27* promoter (*Eco*RI*-Bam*HI fragment), and 1.7-kb tdTomato: *N. crassa* β-tubulin terminator (*Bam*HI*-Hin*dIII fragment) cloned in *Eco*RI*-Hin*dIII sites of pBV141(pBGt). |
| pCK1298 | *PWL2* nuclear reporter vector, consisting of 872-bp *PWL2* promoter (*Eco*RI*-Bam*HI fragment), 0.8-kb EGFP plus NLS (*Bam*HI-*Sal*I fragment) and 0.5-kb *PWL2* 3’-UTR (*Not*I-*Xho*I fragment), cloned in *Eco*RI-*Sal*I sites of pBV1 (pBHt2, Mullins *et al.*, 2001). |
| pCK1528 | Nuclear tdTomato expression binary vector derived from pBV141(pBGt, Kim *et al.*, 2011), consisting of 1.0-kb *RP27* promoter (*Eco*RI*-Bam*HI fragment), and 1.8-kb tdTomato:NLS: *N. crassa* β-tubulin terminator (*Bam*HI*-Hin*dIII fragment) cloned in *Eco*RI*-Hin*dIII sites of pBV141(pBGt). |
| pCK1574 | 872-bp *PWL2* promoter (from pSK1885 (Khang et al., 2010)) into pJET1.2 (Thermo Fisher Scientific). |
| pCK1576 | 1.3-kb sfGFP plus NLS and *PWL2* 3’-UTR into pJET1.2 (Thermo Fisher Scientific). |
| pCK1586 | *PWL2* nuclear reporter vector, consisting of 872-bp *PWL2* promoter (*Eco*RI*-Bam*HI fragment), 1.3-kb sfGFP plus NLS and *PWL2* 3’-UTR (*Bam*HI-*Xba*I fragment), cloned in *Eco*RI-*Xba*I sites of pBV1. |
| pCK1714 | *PWL2* cytoplasmic reporter vector, consisting of 1.7-kb *PWL2* promoter:EGFP (*EcoR*I*-BsrG*I fragment), 0.12-kb protein degradation signal peptide PEST (*Bsr*GI-*Not*I fragment) from pBV118(pd2EGFP-1), and 0.5-kb *PWL2* 3’-UTR (*Not*I-*Xho*I fragment), cloned in *EcoR*I-*Sal*I sites of pBV1. |
| pCK1790 | *PWL2* *Δ* repeats reporter vector, consisting of *PWL2* promoter without 3-repeats (*Eco*RI*-Bam*HI fragment), 1.3-kb sfGFP plus nuclear localization signal and *PWL2* 3’-UTR (*Bam*HI-*Sac*I fragment), cloned in *Eco*RI-*Hin*dIII sites of pBV1. |
| pCK1813 | *PWL2* 1-repeat reporter vector, consisting of *PWL2* promoter with 1-repeat (*Eco*RI*-Bam*HI fragment), 1.3-kb sfGFP plus nuclear localization signal and *PWL2* 3’-UTR (*Bam*HI-*Sac*I fragment), cloned in *Eco*RI-*Hin*dIII sites of pBV1. |
| pCK1822 | *PWL2* reversed repeats reporter vector, consisting of *PWL2* promoter with reversed repeats (*Eco*RI*-Bam*HI fragment), 1.3-kb sfGFP plus nuclear localization signal and *PWL2* 3’-UTR (*Bam*HI-*Sac*I fragment), cloned in *Eco*RI-*Hin*dIII sites of pBV1. |
| pCK1823 | *PWL2* forward repeats reporter vector, consisting of *PWL2* promoter with forward repeats (*Eco*RI*-Bam*HI fragment), 1.3-kb sfGFP plus nuclear localization signal and *PWL2* 3’-UTR (*Bam*HI-*Sac*I fragment), cloned in *Eco*RI-*Hin*dIII sites of pBV1. |
| pCK1883 | *PWL2* reporter vector with repeats at non-original position, consisting of *PWL2* promoter with repeats at non-original position (*Eco*RI*-Bam*HI fragment), 1.3-kb sfGFP plus nuclear localization signal and *PWL2* 3’-UTR (*Bam*HI-*Sac*I fragment), cloned in *Eco*RI-*Hin*dIII sites of pBV1. |
| pCK1887 | *PWL2* 2-repeats reporter vector, consisting of *PWL2* promoter with 2-repeats (*Eco*RI*-Bam*HI fragment), 1.3-kb sfGFP plus nuclear localization signal and *PWL2* 3’-UTR (*Bam*HI-*Sac*I fragment), cloned in *Eco*RI-*Hin*dIII sites of pBV1. |
| pCK1894 | *PWL2* reporter vector with non-specific DNA replacement at repeat position, consisting of *PWL2* promoter with non-specific DNA replacement at repeat position (*Eco*RI*-Bam*HI fragment), 1.3-kb sfGFP plus nuclear localization signal and *PWL2* 3’-UTR (*Bam*HI-*Sac*I fragment), cloned in *Eco*RI-*Hin*dIII sites of pBV1. |
| pCK1905 | *PWL2* 5’-end of one repeat reporter vector, consisting of *PWL2* promoter with 5’-end of one repeat (*Eco*RI*-Bam*HI fragment), 1.3-kb sfGFP plus nuclear localization signal and *PWL2* 3’-UTR (*Bam*HI-*Sac*I fragment), cloned in *Eco*RI-*Hin*dIII sites of pBV1. |
| pCK1922 | *PWL2* 3’-end of one repeat reporter vector, consisting of *PWL2* promoter with 3’-end of one repeat (*Eco*RI*-Bam*HI fragment), 1.3-kb sfGFP plus nuclear localization signal and *PWL2* 3’-UTR (*Bam*HI-*Sac*I fragment), cloned in *Eco*RI-*Hin*dIII sites of pBV1. |
| pCK1969 | *PWL2* cluster II mutation reporter vector, consisting of *PWL2* promoter with cluster II mutation (*Eco*RI*-Bam*HI fragment), 1.3-kb sfGFP plus nuclear localization signal and *PWL2* 3’-UTR (*Bam*HI-*Sac*I fragment), cloned in *Eco*RI-*Hin*dIII sites of pBV1. |
| pCK1975 | *PWL2* cluster I mutation reporter vector, consisting of *PWL2* promoter with cluster I mutation (*Eco*RI*-Bam*HI fragment), 1.3-kb sfGFP plus nuclear localization signal and *PWL2* 3’-UTR (*Bam*HI-*Sac*I fragment), cloned in *Eco*RI-*Hin*dIII sites of pBV1. |
| pCK1988 | *PWL2* cluster III mutation reporter vector, consisting of *PWL2* promoter with cluster III mutation (*Eco*RI*-Bam*HI fragment), 1.3-kb sfGFP plus nuclear localization signal and *PWL2* 3’-UTR (*Bam*HI-*Sac*I fragment), cloned in *Eco*RI-*Hin*dIII sites of pBV1. |
| pCK2004 | *PWL2* 12-bp motif recovery reporter vector, consisting of *PWL2* promoter with a 12-bp motif recovery (*Eco*RI*-Bam*HI fragment), 1.3-kb sfGFP plus nuclear localization signal and *PWL2* 3’-UTR (*Bam*HI-*Sac*I fragment), cloned in *Eco*RI-*Hin*dIII sites of pBV1. |
