# Supplementary material for: Tandem DNA repeats contain cis‐regulatory sequences that activate biotrophy‐specific expression of Magnaporthe effector gene PWL2
Source: Mol Plant Pathol. 2021 Mar 10;22(5):508–21. doi: 10.1111/mpp.13038 (PMC8035637; doi:10.1111/mpp.13038)
Supplement: Supplementary file 14 — TABLE S5 PCR primers used in this study [file MPP-22-508-s014.docx]

**Table S5** PCR primers used in this study.

| **Name** | **Sequence ^a^ (5’-3’)** | **Applications** |
| --- | --- | --- |
| CKP60 | GAATTCGCGTCAGTGAACAAACC | *PWL2* promoter |
| CKP233 | GGATCCCATTTTGAAAGTTTTTAATTTTAAAAAG | *PWL2* promoter |
| CKP267 | GAATCTTTTCACAATGCAGATCAAGGCCCTC | *PWL2* 3’-UTR |
| CKP268 | GAATCTTTTCACAATGGTGAGCAAGGGCGAG | *PWL2* 3’-UTR |
| CKP402 | *CG*GGATCCGTGAGCAAGGGCGAGG | *sfGFP* sequence w/o ATG |
| CKP403 | *GC*TCTAGACTCGAGAAATAGCTTAAAGTAAG | *PWL2* 3’UTR |
| CKP542 | GGTACCAATATATAATTATATATATTAGTACG | *Δ* repeats |
| CKP543 | GGTACCATACAATAAGGGGTTGGCTAATTTATAAG | *Δ* repeats |
| CKP570 | GGTACCTTTTTATTTATGCAAGCTTAC | 2-repeats, 3-repeats |
| CKP571 | GGTACCATGTTTTTTATTCGTCCC | 2-repeats, 3-repeats |
| CKP585 | GAGCTCTTTTTATTTATGCAAGCTTAC | Repeats at non-original position |
| CKP586 | GAATTCATGTTTTTTATTCGTCCC | Repeats at non-original position |
| CKP587 | GGTACCAGGTCGTTCGCTCCAAGC | Non-specific DNA sequence |
| CKP588 | GGTACCCTGTTACCAGTGGCTGCTGC | Non-specific DNA sequence |
| CKP619 | CGAGTAAGCTTGCATAAAT | 5’-end of one repeat |
| CKP620 | ATGCAAGCTTACTCGATACAATAAGGG | 5’-end of one repeat |
| CKP400 | AATATATAATTATATATATTAGTACG | 3’-end of one repeat |
| CKP621 | TAATATATATAATTATATATTCGGATGGGACGAA | 3’-end of one repeat |
| CKP639 | CGCCCGCCCCCAATATATAATTA | Cluster I mutation |
| CKP640 | GGGGGCGGGCGGCAAGCTTACTC | Cluster I mutation |
| CKP641 | CCTAGGTACGCCATAAAAAAATATAT | Cluster II mutation |
| CKP642 | GGCGTACCTAGGACTCGCGGATG | Cluster II mutation |
| CKP643 | ATCTGCCTAGGGCATAAATAAAAA | Cluster III mutation |
| CKP644 | CCTAGGCAGATCGGATGGGACG | Cluster III mutation |
| CKP665J | AAGCTTGCATAAAATATATAATTATATATATTAG | 12-bp motif recovery |
| CKP666J | TTATGCAAGCTTATACAATAAGGGGTTG | 12-bp motif recovery |
| CKP323 | CACTACCTGAGCACCCAGTC | *EGFP* qRT-PCR |
| CKP324 | GAACTCCAGCAGGACCATGT | *EGFP* qRT-PCR |
| CKP327 | GGCGGGTGGACTAACAAACA | *PWL2* qRT-PCR |
| CKP328 | TACCATCCTATCGGGCCCTC | *PWL2* qRT-PCR |
| CKP333 | CGACGTCCGAAAGGATCTGT | *Moactin* qRT-PCR |
| CKP334 | TGCATACGGTCCGAAAGACC | *Moactin* qRT-PCR |
| CKP667J | GGACCTGACGTTCCTGGAC | *MGG_08300* qRT-PCR |
| CKP668J | GGTCAGCTTGAGGACCTTGT | *MGG_08300* qRT-PCR |
| CKP683J | CCGACCGTTATAGCCACTCC | *MGG_01953* qRT-PCR |
| CKP684J | AACCGGGGATTCTGGCATTC | *MGG_01953* qRT-PCR |
| CKP679J | CACTTTGGGAACTGTCGCTG | *Avr-Pik* qRT-PCR |
| CKP680J | TCGGGTACAGGAATACCAGGG | *Avr-Pik* qRT-PCR |
| T7 promoter | TAATACGACTCACTATAGGG | Sequencing confirmation of clones |

^a^Underlined sequences correspond to restriction enzyme sites used for cloning:

*BamH*I (GGATCC), *EcoR*I (GAATTC), *Hind*III (AAGCTT) and *Xba*I (TCTAGA).
